# A cupin domain-containing protein with a quercetinase activity (VdQase) regulates *Verticillium dahliae*'s pathogenicity and contributes to counteracting host defenses

**DOI:** 10.3389/fpls.2015.00440

**Published:** 2015-06-10

**Authors:** Abdelbasset El Hadrami, Md. Rashidul Islam, Lorne R. Adam, Fouad Daayf

**Affiliations:** ^1^Department of Plant Science, University of ManitobaWinnipeg, MB, Canada; ^2^OMEX Agriculture Inc., Oak BluffMB, Canada; ^3^Department of Plant Pathology, Bangladesh Agricultural UniversityMymensingh, Bangladesh

**Keywords:** *Verticillium dahliae* Kleb., Verticillium wilt, biocontrol, plant extract, quercetinase, rutin, phloroglucinol, protocatechuoylphloroglucinol

## Abstract

We previously identified rutin as part of potato root responses to its pathogen *Verticillium dahliae*. Rutin was directly toxic to the pathogen at doses greater than 160 μM, a threshold below which many *V. dahliae* pathogenicity-related genes were up-regulated. We identified and characterized a cupin domain-containing protein (VdQase) with a dioxygenase activity and a potential role in *V. dahliae*-potato interactions. The pathogenicity of VdQase knock-out mutants generated through *Agrobacterium tumefasciens*-mediated transformation was significantly reduced on susceptible potato cultivar Kennebec compared to wild type isolates. Fluorescence microscopy revealed a higher accumulation of flavonols in the stems of infected potatoes and a higher concentration of rutin in the leaves in response to the VdQase mutants as compared to wild type isolates. This, along with the HPLC characterization of high residual and non-utilized quercetin in presence of the knockout mutants, indicates the involvement of VdQase in the catabolism of quercetin and possibly other flavonols *in planta*. Quantification of Salicylic and Jasmonic Acids (SA, JA) in response to the mutants *vs*. wild type isolates revealed involvement of VdQase in the interference with signaling, suggesting a role in pathogenicity. It is hypothesized that the by-product of dioxygenation 2-protocatechuoylphloroglucinolcarboxylic acid, after dissociating into phloroglucinol and protocatechuoyl moieties, becomes a starting point for benzoic acid and SA, thereby interfering with the JA pathway and affecting the interaction outcome. These events may be key factors for *V. dahliae* in countering potato defenses and becoming notorious in the rhizosphere.

## Introduction

Rutin, quercetin, kaempferol, and naringenin are secondary metabolites with a flavonol/none nucleus (i.e., rutin, quercetin, kaempferol, and naringenin; Supplementary Material 1) and often synthesized by higher plants in response to various abiotic and biotic stresses such as UV and microbial diseases, respectively (Harborne and Williams, 2000; Rozema et al., 2002; Grotewold, 2006; Daayf and Lattanzio, 2008). These naturally-occurring anti-oxidants accumulate in various tissues and help prevent oxidative damage (Viornery et al., 2000; Andersen and Markham, 2006; Treutter, 2006). They can be toxic to the pathogen either directly or after oxidization into quinones, thus restricting its growth and development (Bennet and Wallsgrove, 1994; Barry et al., 2002; Treutter, 2006). The quercetin-3-rutinoside *aka* rutin is a flavonol glycoside with a wide presence in fruits and vegetables. In legumes, rutin is released as a signal to initiate nodulation (Peters et al., 1986; Phillips, 1993). It is also released during the interaction with mycorrizhae and after wounding (Oka and Simpson, 1971; Poulin et al., 1993; Lagrange et al., 2001; Martin et al., 2001; Remy et al., 2009).

The co-evolution of pathogens with their hosts helped them tolerate such powerful anti-oxidants through degradation or detoxification. The bacteria colonizing the bowels and intestines of humans and animals are well known for their ability to degrade flavonols such as quercetin (Westlake et al., 1961; Winter et al., 1989, 1991; Kunst et al., 1997; Braune et al., 2001; Krogh et al., 2001; Rose and Fetzner, 2006). The phenomenon is less widespread among fungi and has been documented only in some ascomycetes (*Aspergillus* spp., *Penicillium* spp., *Streptomyces* spp., *Pullularia* sp., *Fusarium oxysporum, Alternaria* spp., *Cephalosporium* spp., and *Diaporthe* spp.) (Westlake et al., 1961; Bartz, 1971; Hund et al., 1999; Steiner et al., 2002; Merkens et al., 2007; Tranchimand et al., 2008) and a few basidiomycetes (*Conidiophora puteana* Karst, *Lentinus lipedius* Fr., *Stereum sanguinolentum* Fr., *Armillaria mellea* Karst, and *Pleurotus ostreatus* Kummer) (Sariaslani and Dalton, 1989). When *Aspergillus flavus* is grown on rutin (quercetin 3-*O*-rhamnoglucoside)-rich media as a singular carbon source, it activates glucosidases and esterase rutinases as well as an inducible quercetinase. The latter is a dioxygenase that is able to cleave two C-C bonds from the heterocyclic ring of quercetin, producing carbon monoxide and 2-protocatechuoylphloroglucinolcarboxylic acid (2-PCPGCA) (Child et al., 1963; Oka et al., 1971). Other fungi such as *Pullularia* spp. (Oka and Simpson, 1971), *Fusarium* spp. (Bartz, 1971), and other *Aspergillus* spp. (Oka et al., 1971) use the same degradation process in generating carbon monoxide while the majority of bacteria do not, due to the reduction of flavonols rather than their oxidation. Three prokaryotes possessing dioxygenases catalyzing the release of carbon monoxide analogously to fungal quercetinases are an exception (Wray and Abeles, 1993; El Hadrami et al., 2009).

*Verticillium dahliae* Kleb. is a soilborne pathogen that causes Verticillium wilt and threatens many important crops including potatoes (Daayf, 2015). This pathogen produces resting structures called microsclerotia that allow it to survive in the soil for several years but also to infect a wide range of hosts. Due to these two features, setting integrated management strategies to control this disease is challenging. In a previous study (Uppal et al., 2007, 2008), we reported on the effectiveness of selected biological treatments in reducing Verticillium wilt in potato plants grown either under controlled conditions or in the field. These included the use of bacterial isolates and extracts from Canada milkvetch. The mechanisms by which such disease protection took place were deemed to be through induced resistance (El Hadrami et al., 2011). Rutin was one of the main secondary metabolites that were induced, with a high accumulation in successful treatments, contrasting with lower induction in the non-effective ones.

The objectives of this study were to evaluate the effect of this differential activation of synthesis and/or accumulation of rutin on *V. dahliae* and to determine the mechanisms involved in the plant protection provided. Knowing the ability of fungi to pre- or post-transcriptionally interfere with host defense-responses and signaling pathways (El Hadrami et al., 2009) and to detoxify accumulated secondary metabolites, we investigated the response of *V. dahliae* to the produced flavonoid. First, we examined *in vitro* the differential expression of selected *V. dahliae* genes activated in response to culture media amended with rutin and related flavonoids. Second, we isolated and characterized the Quercetinase (VdQase) involved in the dioxygenation of quercetin, the aglycone derived from rutin. Then, we generated a knockout mutant to further determine the role of VdQase in *V. dahliae*'s pathogenesis and in countering potatoes defense responses.

## Materials and methods

### The pathogen

Two single spore-*V. dahliae* isolates namely Vd1396-9 (Vd9) and Vd1398-21 (Vd21) were selected for this study as highly aggressive isolates, in addition to their respective generated knockout mutants. The two wild-type isolates were the most aggressive among our collection regularly tested for pathogenicity on potato and/or sunflower (Uppal et al., 2007; Alkher et al., 2009). A weakly aggressive isolate Vs06-14 was also selected for pathogenicity comparison of the mutants. These isolates were grown either on solid culture media [i.e., Potato Dextrose Agar (PDA), water-agar] or on liquid media [i.e., Czapeck-Dox media, inorganic salt media, SDW, complete medium described by Dobinson et al. (1997)] depending on the objectives of each experiment. In addition to these isolates, other *V. dahliae* isolates were used in the *in vitro* and *in planta* tests for utilization of rutin and quercetin.

### Primers design, amplification, and sequencing of quercetinase-coding sequences

Several sets of primers were designed based on known quercetinases from *Penicillium olsonii* (Acc.: EU126643) *Aspergillus japonicas* (Acc.: Q7SIC2), and *Streptomyces* sp. strain FLA (Acc.: CAJ81053) to amplify putative quercetinase-coding sequences in *V. dahliae*. The primers pairs QueVd2F: 5′-CGCGTCTTGAGGTACTGGTT-3′, QueVd2R: 5′-AGAGGCAGATCCGTGTGAGT-3′; QueVd3F: 5′-ACGACGGGCTCGTAATACAC-3′, QueVd3R: 5′-TCCGCTGGTAGCTCTTGTCT-3′; Q2FLAVd2F: 5′-ACACGGTGGTCATGCTTGTA-3′, Q2FLAVd2R: 5′-GGTGCCAGAAACGTCAAAGT-3′ were retained for further investigations. Constitutive 18S RNA gene (Vd18SF: 5′-cggggaggtagtgacgataa-3′; Vd18SR: 5′-cattacggcggtcctagaaa-3′) was used as a control. The primer sets used to amplify the full length genes were FLAVd1F: 5′- CAAAGGACCTGACGGAAGAC -3′, FLAVd1R 5′- TCTCGCACATTCCAGACTTG -3′; FLAVd2F: 5′-ATGACTCAAAAACAGACAGG-3′, FLAVd2R 5′-CTAAGCCACAATCTCGTCAG-3′, and Vd23F: 5′-ATGAGTGTTTCCGTTCACGC-3′and Vd23R1: 5′-CCACTCTCCCCAGACTGAAG-3′. PCR assays were performed using either a C1000™ or MyCycler™ thermal cyclers (BIO-RAD, ON, Canada). The program consisted of 4 min initial denaturation at 94°C followed by 35 cycles with 30 s at 94°C, 30 s at 57°C and 1 min at 72°C and a final extension at 72°C for 10 min. The PCR products were visualized after amplification by electrophoresis on 1.5% agarose gel containing 1% ethidium bromide using a UV-transilluminator (AlphaImager, Alpha Innotech Co., Canada) equipped with a digital camera and a P93D digital monochrome printer (Mitsubishi Digital Electric America Inc.).

### RNA isolation and reverse-transcription of the putative *V. dahliae quercetinase*

Total RNA was extracted using TRIzol® (Invitrogen Co.) according to the manufacturer's recommendations, out of 250 mg FW of mycelia strains grown on liquid media control or amended with 1 ml of commercial standard solutions of the tested compounds (rutin, quercetin, naringenin or phloroglucinol) calibrated at 100 μg/ml in dimethyl sulfoxide (DMSO). RNA quality was verified on 1.5% agarose gel and the concentrations and a Ultrospec 3100 spectrophotometer (Biochrom Ltd., Cambridge, UK) was used to estimate purity based on absorbance at 260 nm and the ratio A260/A280. The first-strand cDNA was synthesized using the M-MLV reverse transcriptase kit from Invitrogen Co. One microliter of total RNA was placed in a nuclease-free 200 μl microcentrifuge PCR tube and mixed with 1 μl of oligo (dT)_12–18_ calibrated at 500 μg ml^−1^ and 1 μl of 10 mM dNTP mix and the volume was adjusted to 12 μl using SD-DEPC-W. The mixture was then heated for 5 min at 65°C and quickly chilled on ice. After a brief centrifugation, the content was mixed with 4 μl of 5X first-strand buffer, 2 μl of 0.1 M DTT and 1 μl of RNaseOUT™ recombinant ribonuclease inhibitor calibrated at 40 U.μl^−1^. The tubes were then gently mixed and incubated for 2 min at 37°C after which 1 μl M-MLV RT (200 U) was added to the contents and re-incubated for 50 min at 37°C. The reaction was last inactivated by heating the mixture for 15 min at 70°C. The synthesized first-strand cDNA was then used as a template for PCR with Q5F/R, QueVd2F/R, QueVd3F/R, Q2FLAVd2F/R primers as described above.

The PCR product amplified was purified using a MinElute® PCR purification Kit according to the manufacturer's instructions (Qiagen Inc.) and sent for sequencing (Macrogen Co., USA). Two independent sets of sequencing were performed out of two independent experiments.

### Sequences analysis

Products of sequencing were examined for any potential misinterpretation and submitted to a BLAST search for potential matches with stored sequences in the GeneBank. The cDNA sequences were also matched against other databases including Cogeme, MIT-Verticillium Genome project (www.broad.mit.edu/annotation/genome/verticillium_dahliae/), FASTA-UK, among others. The predicted proteins based on the best ORFs (NCBI-ORF) were also determined and matched for identities and similarities against most available sequences of quercetinases in the GenBank, EMBL, SwissProt, PIR, and ProDB as well as against known proteins from *V. dahliae* available in NCBI or MIT databases. ClustalW2 (Larkin et al., 2007) was used for DNA and protein sequences alignment to denote conservation, mutation, insertion/deletion events. Phylogenetic dendrograms were generated when necessary using Neighbor Joining algorithm based on percent identity calculated among sequences.

*In silico* analysis and annotation of the protein relied on the use of variety of tools to analyze the sequences (i.e., BLAST, NPS), predict the protein localization (i.e., PRED-CLASS, ProtFun), predict the presence of any putative signal peptide [i.e., SignalP (Bendtsen et al., 2004), SOSUlsignal (Fusetti et al., 2002)]. For topology prediction, secondary structure and generating the 3D structure of the protein the programs ProFunc (Laskowski et al., 2005), Phyre (Kelley and Sternberg, 2009) and WHATIF (Vriend, 1990) were used. The required pdb file required form some of the analysis were generated using either one of these program from the protein sequence or using 3D-JIGSAW (Bates et al., 2001). Superposition of the tested protein model with the reference dioxygenase template from *A. japonicus* or other templates was conducted using the programs SuperPose (Maiti et al., 2004) and WHATIF (Vriend, 1990).

### Assay of the quercetinase activity

Two hundred mg of a freshly grown mycelium from isolates Vd9 and Vd21 were used for proteins extraction in 0.1 M potassium phosphate buffer pH 7.0. The method described by Oka and Simpson (1971) and Oka et al. (1971, 1972) was used to assess the quercetinase activity. This method follows the decrease in *A*_367 nm_ as a result of quercetin breakdown (ε = 20,000 M.cm^−1^) by a known volume of the enzyme added to a mixture consisting of 0.1 M MES buffer at pH 6.0, quercetin and copper. Ten to 20 μl of protein extract were used for the enzymatic assays. One unit of enzyme activity (IU) was set to represent the amount of enzyme required to cleave 1 μM of quercetin per hour under standard conditions. Every assay was repeated five times and the data was averaged after calculation of the activity.

*V. dahliae* quercetinase isozymes were separated by horizontal cellulose acetate gel electrophoresis on 76 × 76 mm CA plates (Helena Laboratories, Beaumont, TX) in 25 mM Tris—192 mM Glycine pH 8.5 buffer. Fifty to 100 μl of protein extract were used for the assays. Electrophoresis gels were run at 175–200 V for 15–20 min till blue dye consisting of 0.25% bromophenol blue, 25% Ficoll reached the end of the gel. After a complete run, isozymes were stained in 0.1 M MES buffer, pH 6.0 mixture containing 60 μl of quercetin, 120 μl of CuSO_4_, 80 μl of 10 mM MTT, 80 μl of 0.2% PMS blended with 1.6% agar. The cellulose acetate plates were then left in the staining solution at room temperature. When necessary, the gel was fixed in water-methanol-acetic acid (5:5:1, v/v/v) for 5 min.

### Nucleic acid manipulations

#### Cloning and targeted disruption of *V. dahliae VdQase*

A 3.8 kb *VdQase* coding sequence was amplified from the genomic DNA of Vd9 (Vd1396-9, a highly aggressive isolates of *V. dahliae* on potato using primers VdQase-FP (5′-AAGCTT ATGAGTGTTTCCGTTCACGC-3′) and VdQase-RP (5′-AAGCTT TCATCCATTGCTGGCCTCGT-3′) (underlined sequences indicate the *Hind*III restriction site in both primers) using the Phusion High Fidelity DNA polymerase (Invitrogen). After an initial denaturation at 98°C for 30 s the PCR conditions were denaturation at 98°C for 10 s, annealing at 68°C for 45 s and elongation at 72°C for 2:30 min for 35 cycles followed by a final extension at 72°C for 10 min. The fragment was then cloned in pGEM T-Easy Vector (Promega) and confirmed by sequencing using M13 forward and reverse primers. The *Hind*III VdQase fragment was then sub-cloned at the same site of the binary plasmid pDHt [41] and confirmed by PCR, restriction digestion and sequencing. The cloned gene was then mutagenized using the EZ::TN system (Epicentre Technologies, Madison, WI) as described by Dobinson et al. (2004). The plasmids mutagenized was then electroporated into *Escherichia coli* strain DH10B (Invitrogen) and the EZ::TN insertion into the *VdQase* coding sequence were verified by PCR amplification using the plasmid from the chloramphenicol resistant clones as template with VdQase specific primers as mentioned above. The positive clones were then sequenced to determine the EZ::TN insertion sites in the VdQase coding region using the pMOD-2 forward (5′-GCCAACGACTACGCACTAGCCAAC-3′) and reverse (5′-GAGCCAATATGCGAGAACACCCGAGAA-3′) sequencing primers (Epicentre Technologies). The selected sequenced pDHt plasmid that contains the mutagenized VdQase gene was then transformed into *Agrobacterium tumefaciens* AGL-1 strains by electroporation using Gene Pulser (BioRad). The clones were then confirmed by PCR using the primers and conditions mentioned above.

#### Production on VdQase knockout mutants

Highly aggressive isolate Vd9 was selected as a wild type to generate the VdQase knockout mutant and conduct functional analyses. ATMT of *V. dahliae* was carried out using *Agrobacterium tumefasciens*-mediated transformation (ATMT) as described by Dobinson et al. (2004) and Mullins et al. (2001) with slight modifications. *V. dahliae* wild type strain was grown on PDA for at least 1 week and conidia were harvested *prior to* co-cultivation. Timentin (200 μg/mL) was used instead of Moxalactum in the selection medium. Hygromycin B-resistant transformants were cultured in CM medium containing hygromycin in 24-well cell culture plates. Three to 5 days after, samples of each mutant culture were transferred onto PDA and single spore isolates were selected and re-cultured to produce mycelia and spores.

### Confirmation of mutant transformants

The transformants were screened using PCR, southern blot hybridization and RT-PCR. PCR was used to confirm the gene-insertion mutants at the specific location of T-DNA integration using primers VdQase Upstream FP (5′-TGGTGTTGTTGCTCGGCATTTCGT-3′), a primer that was designed few hundreds base pairs upstream of the start codon of VdQase, and the specific reverse primer for VdQase gene mentioned above. For southern hybridization, genomic DNA was extracted from the wild type and the mutant isolates. The probe hygromycin B was amplified from the pSK846 using primers HyF (5′-TCAGCTTCGATGTAGGAGGG-3′) and HyR (5′-TTCTACACAGCCATCGGTCC-3′) and was labeled according to the GE Healthcare instruction manual. For blotting, genomic DNA (20 μg) of both the wild type and mutant transformants was digested with *EcoR*I (Promega) and separated on agarose gel before being transferred onto Amersham Hybond N^+^ membranes (GE Healthcare). The hybridizations and signals detection on the Kodak X-Omat film as described in the Alkhos labeling kit (GE Health Care). For RT-PCR, total RNA was extracted from the wild type and the transformants, grown on CDX and CM media, respectively, using TRIzol reagent (Invitrogen). c-DNA was synthesized as described above using the primer pair VdQase RT FP (5′-GGCAAGGACTGGAGGAGTTGATT-3′) and VdQase RT RP (5′-GTGATTGCCATT GCCGACGGTA-3). PCR conditions were 95°C for 3 min initial denaturation followed by 35 cycles of denaturation at 95°C for 30 s, annealing at 60°C for 30 s, and extension at 72°C for 1 min and a final extension 72°C for 5 min.

### Pathogenicity testing

The single spore *V. dahliae* wild type and mutant isolates were maintained on PDA at 20 ± 2°C until used. Pathogenicity tests were conducted in triplicates as described by Alkher et al. (2009) and the whole experiment was repeated three times. Briefly, Four-week-old potato plants were used for inoculations. Inoculum of each *V. dahliae* isolates were prepared from single-spore cultures grown on PDA for 2 weeks at 20°C (Fisher Scientific Incubator, Model 146E). Conidial suspensions were prepared to a final concentration of 1 × 10^6^ conidia/mL. The plants were inoculated via “root dip” inoculation (Alkher et al., 2009). Briefly, the soil was gently washed from the uprooted potato plant roots with water followed by trimming the root tips with scissors. The root systems were then immersed in the conidial suspensions for 1 min before being transplanted. The plants for the wounded control treatment had their root tips cut before immersion in sterile distilled water (SDW). The inoculation experiments were conducted with three replications. In *Arabidopsis thaliana*, the inoculation was conducted with slight modifications.

### *In vitro* and *in planta* utilization of rutin and quercetin

Rutin and quercetin *in vitro* utilization by the wild type and VdQase mutants was assessed as previously described by El Hadrami et al. (2011) with a slight modification. Quantification was conducted using HPLC and the data was expressed as percentage of the untreated control for the *in vitro* tests. All analyses were conducted in triplicates and repeated at least twice. The selected isolates (Vd9, Vd9+25-5, Vd9+25-7, and Vs06-14) were grown in 100 ml of Czapeck-Dox (CDX) liquid medium (Hurst et al., 2002), either non-amended, or amended with 1 ml of commercial standard solutions of the tested compounds calibrated at 100 μg/ml in dimethyl sulfoxide (DMSO). Media amended only with 1 ml of DMSO were used as controls. The inoculated media as well as the controls were kept at room temperature on a shaker (Edison, NJ, USA) in the dark at 120 rpm. Samples consisting of 1-ml solutions were taken at 0, 3, 5, 8, 15, 21, and 28 days after transfer. They were immediately analyzed by HPLC or kept at −20°C until needed. Identification of each compound or its product of degradation was based on characteristics including absorption spectra, retention times, comparison and co-elution with commercial standards and on TLC and HPLC. Quantitative analyses were performed by HPLC using the peak area with reference to a standard curve made for each commercial standard (Sigma-Aldrich Co.). Three replicates per treatment x isolate combination were considered for this experiment. Quantifications of the added compounds and their products of degradation were performed in triplicate and the data are reported as the recorded average values.

### Determination of SA and JA levels

Leaf samples were collected from potato plants inoculated with wild type and mutant isolates at 2, 3 and 4 w.p.i. for the determination of SA and JA. For SA, 500 mg of tissues were reduced to powder under liquid nitrogen and suspended in 500 μl of 80% methanol amended with 10 μl of *O*-anisic acid (Sigma-Aldrich) calibrated at 250 ng/ml as an internal standard (Meuwly and Métraux, 1993). The samples were transferred overnight onto a shaker placed at 4°C then centrifuged at 7000 *g* for 5 min. After evaporation of the methanol fraction, free SA was extracted using ethyl acetate (v/v) and re-suspended in pure methanol. For the extraction of bound SA, the aqueous phases were hydrolyzed with 4N HCl at 100°C for 2 h followed by two times extraction with ethyl acetate. After evaporation of the organic phases, the residues were re-suspended in pure methanol. Samples were run on a Waters Alliance 2695 HPLC equipped with at Waters 996 PDA and W2475 Fluorescence detectors. Samples were injected onto an Agilent Poroshell 120 EC-C18 column (4.6 × 100 mm, 2.7 μm) with mobile phase A (water with 0.1% *O-*phosphoric acid) and B (Acetonitrile) at a flow rate of 1 ml/min. The following gradient was used for the elution: Time (min) – %A – %B 0-100-0; 2-95-5; 5.6-90-10; 8-80-20; 12-65-35; 17.2-50-50; 19.2-25-75; 22-0-100; 24.8-100-0; 26-100-0. The W2475 fluorescence detector was set for 290 nm excitation wavelength and an emission scanning from 300 to 500 nm. SA was detected at 390 nm while the *O*-anisic acid internal standard was measured at 350 nm. Molar concentration of SA was adjusted by expected molar concentration of the internal standard.

JA was quantified using (±)-9,10-dihydrojasmonic acid DHJA (OlChemIm Ltd. Czech Republic) as an internal standard. For the extraction, 500 mg of tissues were reduced to powder using liquid nitrogen and suspended in 5 ml of extraction solvent (HCl:2-propanol: H_2_O in a ratio of 2:1:0.002) containing 50 μl of DHJA calibrated at 1 μg/ml. The mixtures were shaken for 30 min at 4°C then mixed with 7 ml of dichloromethane. After centrifugation at 7000 g for 5 min, the supernatants were evaporated to dryness and re-suspended in 50% methanol. Samples were injected onto an Acquity UPLC equipped with a Micromass Quattro micro LC/MS/MS in ESI mode. Samples were injected onto a Phenomenex Kinetex (100 × 2.1 mm 1.7 μm) column with mobile phase A (water containing 0.1% formic acid) and B (methanol containing 0.1% formic acid) at 0.22 ml/min with the following gradient: Time (min)-%A-%B: 0-70-30; 2-70-30; 12-0-100; 13-0-100; 15-70-30. JA and DHJA were quantified as a peak area of MRM data.

### Flavonoid detection by fluorescence microscopy

*In situ* detection of flavonoids, including flavonols, was done using fluorescence microscopy. Stem samples were taken 28 dpi from the bottom, middle and top parts of the tested potato plants. Free hand cross-sections were immersed in NEU reagent (1% methanolic 2-aminoethyl diphenylborinate, Sigma-Aldrich D9754) before observation under a fluorescence microscope (Vanguard Microscopes, Model 1486FL, VEE GEE Scientific Inc., Kirkland, WA USA) with filter set Exciter, Dichroic and Emitter wavelengths of 365, 400, and 535 nm, respectively. The NEU reagent reveals the presence of many flavonoids in bright yellow or orange under UV-366 nm.

### Experimental design and data analysis

All experimental trials were done in triplicates and repeated at least once over time unless otherwise stated. Quantitative data was submitted to ANOVA analysis using Statistica software (Statsoft Inc., 1999). The pairwise comparison of the means was performed according to using Newman-Keuls test at *P* < 0.05. Quercetinase activity was based on five independent assays performed for each treatment. To determine the enzymatic activity data points were collected and used in a linear regression of absorbance decrease at 367 nm (*A*_367 nm_) over time.

Sequences alignment was conducted using ClustalW2 (Larkin et al., 2007) and dendrograms were generated using algorithms such as PRRN (progressive pairwise alignment and iterative refinement; Gotoh, 1999) and multiple sequence alignment based on fast Fourier transform, MAFFT (Katoh et al., 2002). Neighbor-Joining algorithm was used to generate phylogenetic dengdrograms based on the percent identity among the compared sequences.

Other programs used during the *in silico* characterization of the protein generated specific statistical parameters to assess the robustness of the predictions.

## Results

### Gene expression in *V. dahliae* grown in flavonol-amended liquid media

**Figure 1:**
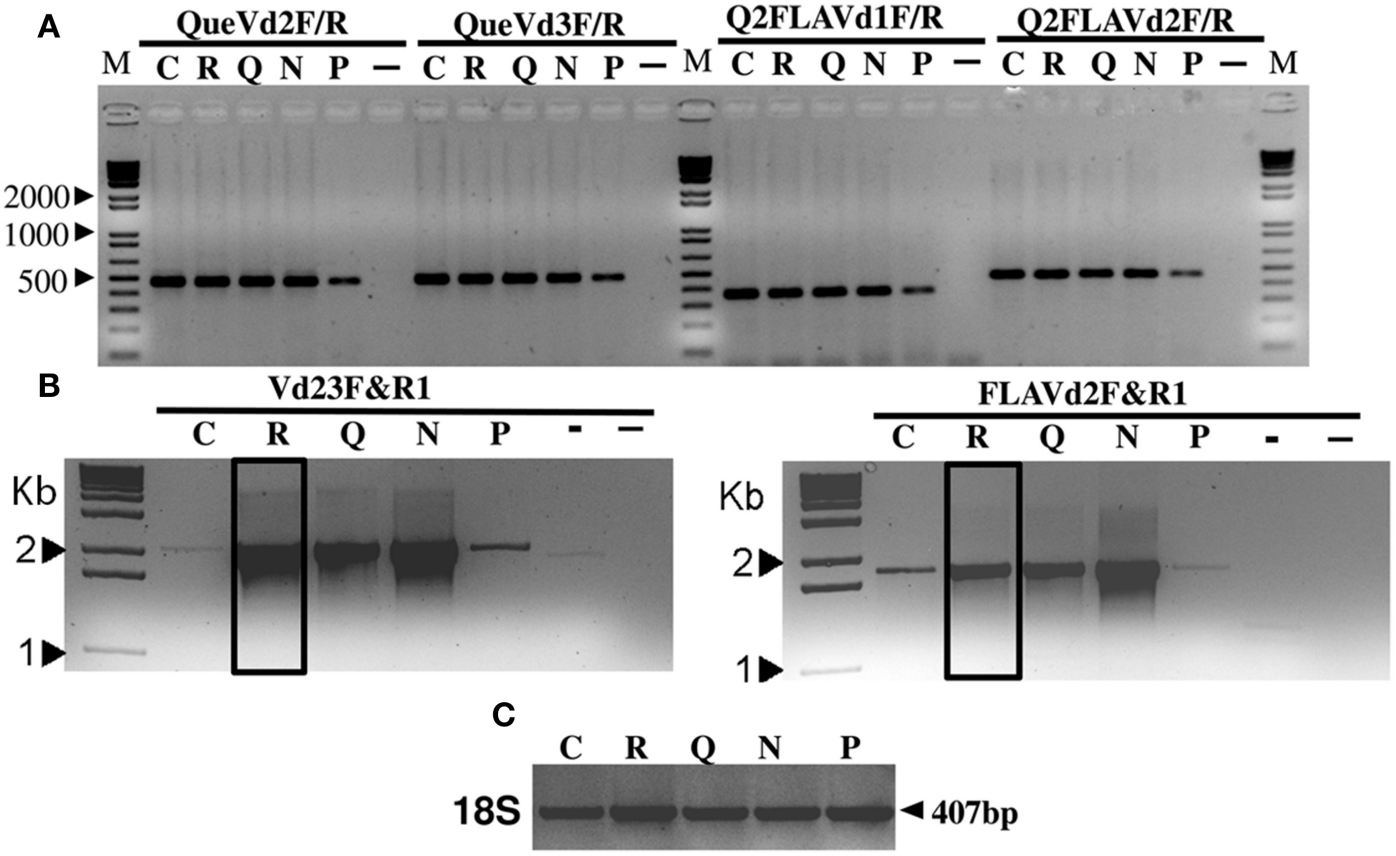
**RT-PCR products amplified with primer pairs QueVd2F/R, QueVd3F/R, Q2FLAVd1F/R, and Q2FLAVd2F/R out of mRNA isolated from** ***V. dahliae*** **isolate Vd9 grown on liquid media control (C) or amended with rutin (R), quercetin (Q), naringenin (N), or phloroglucinol (P)**. (-): negative control. (–): double negative control using plant RNA. Similar profiles were detected using isolate Vd21 and were not included for space convenience. **(A)** represents the initial amplification of the transcripts with various sets of primers while **(B)** represents the relative abundance of the full length transcripts of each putative VdQase gene amplified using Vd23F/R1 and FLAVd2F/R1 primer sets. **(C)** represents the amplification of 18S rRNA used as reference gene.

Primer sets QueVd2F/R, QueVd3F/R, Q2FLAVd1F/R, and Q2FLAVd2F/R were designed based on known quercetinases from *P. olsonii, Aspergillus japonicus* and *Streptomyces* sp. strain FLA, respectively, to amplify putative quercetinase-coding sequences in *V. dahliae*. RT-PCR products amplified with these primer sets (Figure 1A) were sequenced and BLAST-searched (Altschul et al., 1997) against the annotated *V. dahliae* genome available on MIT website (www.broadmit.edu/). The analysis showed 85–100% identity of the sequences with cupin 1, Zn finger C2H2 transcripts fully sequenced and annotated with no assigned function, and located on chromosome 3 of the two reference isolates in the database (Table 1). Using the original quercetinase sequences from *P. olsonii* and *A. japonicus* in the BLAST-search revealed 82% identity with the same cupin domain-containing protein matching the sequences amplified with QueVd2F/R and QueVd3F/R. Against *V. dahliae* VdLs.17 ORF VDAG_02532.1 the parameters of the match were: Score = 87.81 bits (216); Expect = 8.88257e^−18^; Identities = 82/342 (23%); Positives = 143/342 (41%). The ORF was then named VdQase.

**Table 1:** **Matches of the amplified** ***V. dahliae*** **sequences against annotated** ***V. dahliae*** **genome available through MIT website (www.broadmit.edu/)**.

**Primers set and target**	**Score (Bits)**	**Expect**	**Identities (%)**	**Positives (%)**
**QueVd2 F/R**				
*V. dahliae* VdLs.17: VDAG_02532.1: cupin domain-containing protein	263.462 (672)	0.0	127/127 (100%)	127/127 (100%)
*V. albo-atrum* VaMs.102: VDBG_02075.1: conserved hypothetical protein	227.254 (578)	0.0	112/127 (88%)	114/127 (89%)
**QueVd3 F/R**				
*V. dahliae* VdLs.17: VDAG_02532.1: cupin domain-containing protein	288.5 (737)	0.0	144/148 (97%)	145/148 (97%)
*V. albo-atrum* VaMs.102: VDBG_02075.1: conserved hypothetical protein	239.58 (610)	0.0	122/147 (82%)	130/147 (88%)
**Q2FLAVd2 F/R**				
*V. dahliae* VdLs.17: VDAG_02534.1: conserved hypothetical protein	172.17 (435)	3.9e^−44^	103/103 (100%)	103/103 (100%)
*V. albo-atrum* VaMs.102: VDBG_02078.1: conserved hypothetical protein	93.2041 (230)	2.3e^−20^	60/104 (57%)	69/104 (66%)

BLAST-search of the sequenced transcripts amplified using primer sets QueVd2F/R, QueVd3F/R against other databases revealed homologies with conserved transcription and DNA binding factors that are often involved in metal binding (i.e., copper; Acc. emb|CAP79125.1) or in the protection against organic peroxides (i.e., AhpA protein; Acc. gb|AAT02761.1) (Table 2). Transcripts amplified using primer set Q2FLAVd2F/R showed similarities with membrane permeases, sugar transporters/permeases, and structural and transmembrane proteins (Table 2). Interestingly, known quercetinases from *P. olsonii* and *A. japonicus* showed similarities with these same proteins.

**Table 2:** **Matches of the amplified** ***V. dahliae*** **sequences against NCBI GenBank**.

**Matching Accession**	**Putative function**	**Organism**	**Score (Bits)**	***E* value**
**Vd21Q1-QUEVd2F/R**				
ref|XP_389742.1| ref|XP_389223.1| ref|XP_001909254.1| ref|XP_389219.1| ref|XP_001933004.1| ref|XP_001932443.1| ref|XP_001596875.1| gb|EDP56789.1| ref|XP_752922.1| ref|XP_001223987.1| ref|XP_001796737.1| ref|XP_001555211.1| ref|XP_001264194.1| emb|CAP79125.1| emb|CAP97951.1| ref|XP_682597.1| ref|XP_388085.1| ref|XP_001273098.1| gb|AAT02761.1| ref|XP_659624.1|	hypothetical protein FG09566.1 hypothetical protein FG09047.1 unnamed protein product hypothetical protein FG09043.1 conserved hypothetical protein conserved hypothetical protein hypothetical protein SS1G_03098 homeobox C2H2 transcription factor, putative homeobox and C2H2 transcription factor hypothetical protein CHGG_04773 hypothetical protein SNOG_06363 hypothetical protein BC1G_06341 homeobox C2H2 transcription factor, putative Pc06g01320, similar to copper homeostasis Pc22g06630, DNA binding domain hypothetical protein AN9328.2 hypothetical protein FG07909.1 homeobox C2H2 transcription factor, putative AhpA, protection against organic peroxides hypothetical protein AN2020.2	*Gibberella zeae* PH-1 *Gibberella zeae* PH-1 *Podospora anserina* *Gibberella zeae* PH-1 *Pyrenophora tritici-repentis* Pt-1C-BFP *Pyrenophora tritici-repentis* Pt-1C-BFP *Sclerotinia sclerotiorum* 1980 *Aspergillus fumigatus* A1163 *Aspergillus fumigatus* Af293 *Chaetomium globosum* CBS 148.51 *Phaeosphaeria nodorum* SN15 *Botryotinia fuckeliana* B05.10 *Neosartorya fischeri* NRRL 181 *Penicillium chrysogenum* Wisconsin 54-1255 *Penicillium chrysogenum* Wisconsin 54-1255 *Aspergillus nidulans* FGSC A4 *Gibberella zeae* PH-1 *Aspergillus clavatus* NRRL 1 *Emericella nidulans* *Aspergillus nidulans* FGSC A4	100 99.8 99.4 99.0 96.7 96.3 95.5 95.1 95.1 94.7 94.4 93.6 93.2 78.2 77.8 77.0 75.1 74.7 53.1 53.1	6e-20 7e-20 1e-19 1e-19 6e-19 8e-19 1e-18 2e-18 2e-18 2e-18 3e-18 5e-18 7e-18 2e-13 3e-13 5e-13 2e-12 3e-12 8e-06 8e-06
**Vd21Q1-QUEVd3F/R**				
ref|XP_391592.1| ref|XP_389742.1| ref|XP_388085.1| ref|XP_961794.1| ref|XP_001932443.1| ref|XP_389223.1| ref|XP_001800433.1| ref|XP_001223987.1| ref|XP_363804.2| ref|XP_001555211.1| ref|XP_001596875.1|	hypothetical protein FG11416.1 hypothetical protein FG09566.1 hypothetical protein FG07909.1 hypothetical protein NCU05257 conserved hypothetical protein hypothetical protein FG09047.1 hypothetical protein SNOG_10151 hypothetical protein CHGG_04773 hypothetical protein MGG_01730 hypothetical protein BC1G_06341 hypothetical protein SS1G_03098	*Gibberella zeae* PH-1 *Gibberella zeae* PH-1 *Gibberella zeae* PH-1 *Neurospora crassa* OR74A *Pyrenophora tritici-repentis* Pt-1C-BFP *Gibberella zeae* PH-1 *Phaeosphaeria nodorum* SN15 *Chaetomium globosum* CBS 148.51 *Magnaporthe grisea* 70-15 *Botryotinia fuckeliana* B05.10 *Sclerotinia sclerotiorum* 1980	75.5 68.9 67.8 62.8 62.0 59.3 57.0 57.0 55.8 38.1 37.4	2e-12 1e-10 3e-10 1e-08 2e-08 1e-07 6e-07 6e-07 1e-06 0.27 0.45
**Vd21Q1-FLAQ2**				
ref|XP_384886.1| ref|XP_001402423.1| emb|CAP98741.1| ref|XP_001382383.1| ref|XP_722051.1| dbj|BAG11496.1| emb|CAX41832.1| ref|XP_001385456.1| ref|XP_001526533.1| ref|XP_001262269.1| ref|NP_011805.1| ref|XP_747524.1| gb|EEH07499.1| emb|CAB46745.1| gb|EED13883.1| dbj|BAG11506.1| gb|EED14705.1| gb|EED14397.1| gb|EDP49077.1| ref|XP_572882.1| gb|EED24629.1| ref|XP_568347.1| gb|EED53463.1| gb|ABV21349.1| ref|XP_001261420.1| ref|XP_571538.1| emb|CAD36557.1| ref|XP_571470.1| ref|XP_001258307.1|	hypothetical protein FG04710.1 hypothetical protein An10g00300 Pc22g14530, secondary transporter maltose permease potential maltose permease alpha-glucoside permease alpha-glucoside transporter, putative maltose permease hypothetical protein LELG_01361 MFS maltose permease, putative Maltose permease, inducible high-affinity MFS maltose permease trehalose transporter maltose permease MFS maltose permease, putative alpha-glucoside permease sugar transporter, putative sugar transporter, putative MFS sugar transporter, putative sugar transporter hexose carrier protein, putative trehalose transport-related protein sugar transporter, putative maltose transporter maltose permease trehalose transport-related protein maltotriose symporter alpha-glucoside transport-related protein maltose permease	*Gibberella zeae* PH-1 *Aspergillus niger* CBS 513.88 *Penicillium* *chrysogenum* Wisconsin 54-1255 *Pichia stipitis* CBS 6054 *Candida albicans* SC5314 *Saccharomyces cerevisiae* *Candida dubliniensis* CD36 *Pichia stipitis* CBS 6054 *Lodderomyces elongisporus* NRRL YB-4239 *Neosartorya fischeri* NRRL 181 *Saccharomyces cerevisiae* *Aspergillus fumigatus* Af293 *Ajellomyces* *capsulatus* G186AR *Kluyveromyces lactis* *Talaromyces stipitatus* ATCC 10500 *Saccharomyces cerevisiae* *Talaromyces stipitatus* ATCC 10500 *Talaromyces stipitatus* ATCC 10500 *Aspergillus fumigatus* A1163 *Cryptococcus neoformans* JEC21 *Talaromyces stipitatus* ATCC 10500 *Cryptococcus neoformans* JEC21 *Aspergillus flavus* NRRL3357 *Saccharomyces* *pastorianus* *Neosartorya fischeri* NRRL 181 *Cryptococcus* *neoformans* JEC21 *Saccharomyces pastorianus* *Cryptococcus neoformans* JEC21 *Neosartorya fischeri* NRRL 181	114 76.3 75.5 70.5 70.5 69.7 68.9 68.2 67.0 67.0 66.6 65.5 65.1 64.7 63.9 63.9 63.5 62.8 62.8 62.0 61.6 60.8 60.5 60.5 60.5 60.5 60.5 60.5 59.3	4e-24 9e-13 2e-12 5e-11 5e-11 8e-11 1e-10 2e-10 5e-10 5e-10 7e-10 2e-09 2e-09 3e-09 5e-09 5e-09 6e-09 1e-08 1e-08 2e-08 2e-08 4e-08 5e-08 5e-08 5e-08 5e-08 5e-08 5e-08 1e-07

The percent identity of the amplified DNA sequences from both isolates Vd9 and Vd21 with well characterized quercetinases from *P. olsonii* and *Streptomyces* sp. strain FLA averaged 50% (Table 3).

Table 3**Percent identities of putative quercetinases from two** ***V. dahliae*** **isolates (Vd9 and Vd21) amplified using primers' sets QUEVd2F/R and QUEVd3F/R as well as FLAQ2F/R with known quercetinases from** ***P. olsonii*** **(A) or** ***Streptomyces*** **sp. FLA strain (B)**.

**Table:** 

**(A)**													
	**Vd9R2QU EVd2R**	**Vd9Q1QU EVd2R**	**Vd9N2QU EVd2R**	**Vd21R2Q UEVd2**	**Vd21Q1Q UEVd2**	**Vd21N1Q UEVd2**	**Vd9R2-QUEVd3**	**Vd9Q1-QUEVd3**	**Vd9N2-QUEVd3**	**Vd21R2Q UEVd3**	**Vd21Q1Q UEVd3**	**Vd21N1Q UEVd3**	**Que–polsonii**
Vd9R2-QUEVd2R	***100***												
Vd9Q1-QUEVd2R	98.45	***100***											
Vd9N2-QUEVd2R	97.33	97.78	***100***										
Vd21R2-QUEVd2R	98.89	98.90	97.78	***100***									
Vd21Q1-QUEVd2R	99.32	99.77	98.19	99.32	99.32	***100***							
Vd21N1-QUEVd2R	99.32	99.77	98.19	99.32	99.32	***100***							
Vd9R2–QUEVd3R	50.12	50.00	51.04	50.12	49.19	49.88	***100***						
Vd9Q1–QUEVd3R	52.03	51.90	51.77	52.03	51.07	51.78	97.79	***100***					
Vd9N2–QUEVd3R	52.12	52.00	51.87	52.12	51.18	51.88	97.57	99.56	***100***				
Vd21R2-QUEVd3R	51.25	50.88	51.29	51.25	51.28	50.77	98.41	99.54	99.32	***100***			
Vd21Q1-QUEVd3R	51.75	51.36	51.50	51.34	51.26	51.24	98.02	99.78	99.56	99.55	***100***		
Vd21N1-QUEVd3R	52.02	51.78	50.69	52.02	51.92	50.92	98.65	99.09	98.87	99.33	99.11	***100***	
Que-polsonii	50.85	51.65	50.57	49.73	50.09	49.27	51.70	51.51	49.72	48.76	51.59	51.62	***100***

**Table:** 

**(B)**							
	**Vd9R2-FLAQ2**	**Vd9Q1-FLAQ2**	**Vd9N2-FLAQ2**	**Vd9R2-FLAQ2**	**Vd9Q1-FLAQ2**	**Vd9N1-FLAQ2**	**QueDStrepFLA**
Vd9R2-FLAQ2	***100***						
Vd9Q1-FLAQ2	98.74	***100***					
Vd9N2-FLAQ2	99.05	98.74	***100***				
Vd9R2-FLAQ2	99.37	98.75	99.05	***100***			
Vd9Q1-FLAQ2	99.05	99.69	99.05	99.07	***100***		
Vd9N1-FLAQ2	99.05	99.38	99.05	99.07	100	***100***	
QueDStrepFLA	54.19	53.57	52.91	54.14	53.20	53.18	***100***

*The isolates were grown on liquid media amended with either rutin (Rx), quercetin (Qx) or naringenin (Nx). The ‘x’ represents the flask number where the isolates were grown. Bold italic depicts 100% identity*.

The incubation of the tested *V. dahliae* isolates (Vd9 and Vd21) in presence of rutin, quercetin and naringenin induced a substantial accumulation of VdQase transcripts as compared to the untreated control or in mycelia incubated in presence of phloroglucinol (Figure 1B). This allowed us to amplify homolog genes' full-length sequences of ~1.5 and 2.2 Kb with Vd23F/R1 and FLAVd2F/R1 primer pairs, respectively. No effect of the treatments was recorded on the expression of the 18S rRNA used as a reference gene (Figure 1C). The analysis of these transcripts using Phyre (Kelley and Sternberg, 2009) and ConFunc (Wass and Sternberg, 2008) revealed a strong homology and 24–26% identity with RmlC-like Cupin from *A. japonicus* (SCOP Code: d1juha_) or *Bacillus subtilis* (SCOP Code: d1y3ta1); both known as Quercetin 2,3-dioxygenase-like proteins (Supplementary Material Xls file 1).

### *In silico* annotation, structure identification and functional analysis of the first quercetinase in *v. dahliae* (VdQase)

**Figure 2:**
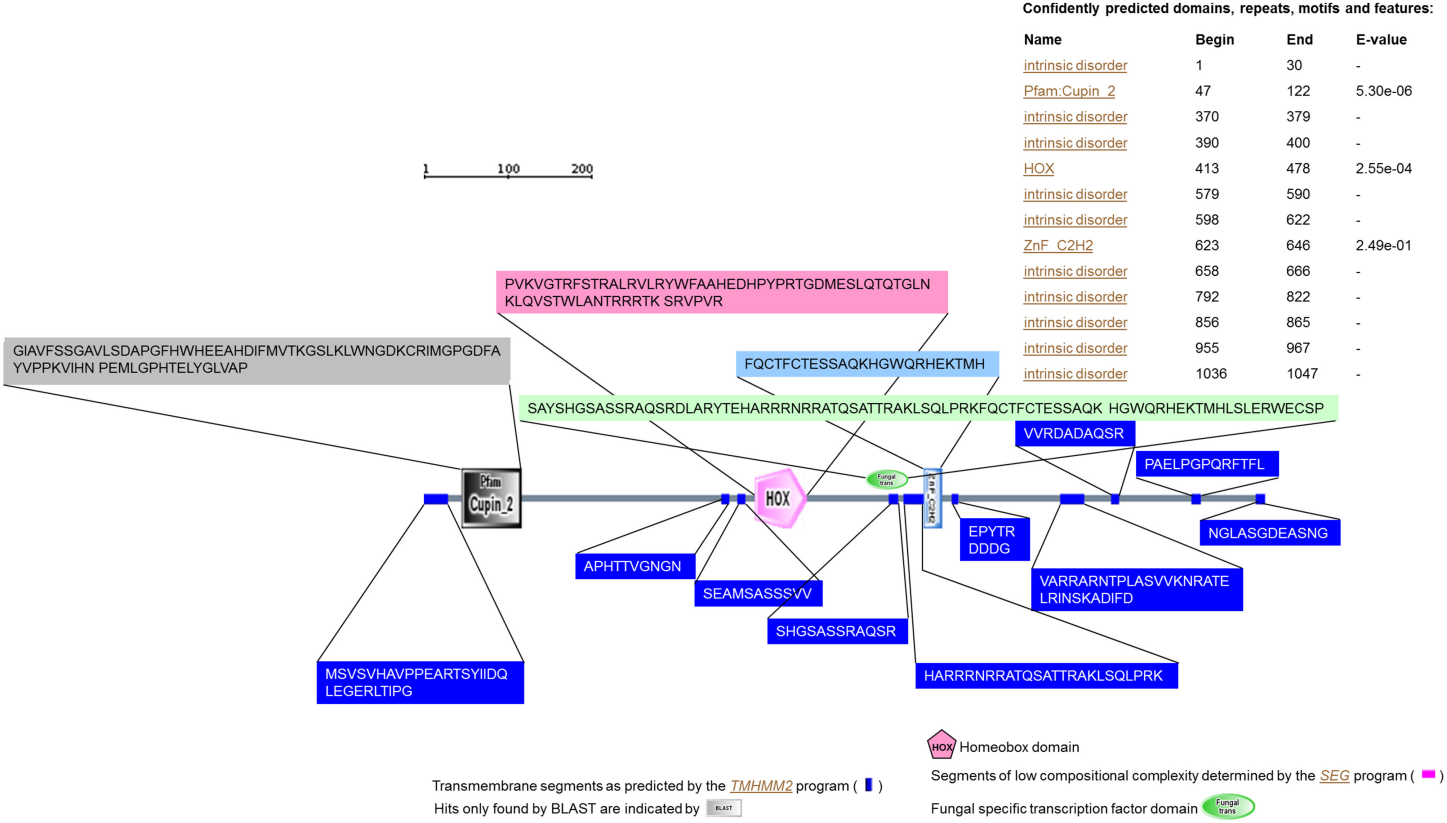
**Annotation of the quercetinase from** ***V. dahliae*** **using SMART program**. Using TMHMM2 program (Katoh et al., 2002) the annotation of the protein revealed the presence of several transmembrane segments. Using PROSCAN (Gattiker et al., 2002) several motifs were detected based on 100% identity or a percent similarity higher than 80%. These motifs included an *N*-glycosylation site (N-{P}-[ST]-{P}), a cAMP- and cGMP-dependent protein kinase phosphorylation site ([RK](2)-x-[ST]), a protein kinase C phosphorylation site ([ST]-x-[RK]), domain signatures for hemopexin ([LIFAT]-{IL}-x(2)-W-x(2,3)-[PE]-x-{VF}-[LIVMFY]-[DENQS]-[STA]-[AV]-[LIVMFY]), “Homeobox” ([LIVMFYG]-[ASLVR]-x(2)-[LIVMSTACN]-x-[LIVM]-{Y}-x(2)-{L}-[LIV]-[RKNQESTAIY]-[LIVFSTNKH]-W-[FYVC]-x-[NDQTAH]-x(5)-[RKNAIMW]), Zinc finger C2H2 type (C-x(2,4)-C-x(3)-[LIVMFYWC]-x(8)-H-x(3,5)-H), and bZIP ([KR]-x(1,3)-[RKSAQ]-N-{VL}-x-[SAQ](2)-{L}-[RKTAENQ]-x-R-{S}-[RK]). Besides, the same analysis confirmed a signature for ABC transporters family ([LIVMFYC]-[SA]-[SAPGLVFYKQH]-G-[DENQMW]-[KRQASPCLIMFW]-[KRNQSTAVM]-[KRACLVM]-[LIVMFYPAN]-{PHY}-[LIVMFW]-[SAGCLIVP]-{FYWHP}-{KRHP}-[LIVMFYWSTA]).

The predicted protein from transcript homolog of VDAG_02532.1 (www.broadmit.edu/) had 1047 residues. No signal peptide was predicted using either SignalP (Bendtsen et al., 2004) or SOSUIsignal (Gomi et al., 2004) programs. Residues comprised between 2 and 323 were revealed to be encoded by the quercetinase gene using ProFunc program (Laskowski et al., 2005). Using SCRATCH program (Cheng et al., 2005), three domains were predicted. Domain 1 spans from residue 1 to 559; domain 2 ranges from 560 to 769 and the third domain contains residues 770-1047. Sixteen cysteines were detected and six disulfide bonds were predicted (Cys1-Cys2: 340-361; 220-252; 824-854; 625-628; 88-995; 654-686). Annotation using various programs such as SMART, SWISS-Model, TMHMM2 and PROSCAN (Gattiker et al., 2002; Katoh et al., 2002; Rozema et al., 2002; Schwede et al., 2003; Letunic et al., 2004; Larkin et al., 2007) allowed for the prediction with confidence of several conserved motifs including a Cupin 2, a homeodomain (HOX) and a ZnF_C2H2 as well as a fungal-specific transcription factor (Figure 2).

Using 3D-JIGSAW (Bates et al., 2001), a pdb signature was generated for the characterized protein. The analysis of this signature using the program ProFunc (Laskowski et al., 2005) allowed for the characterization of the topology and the secondary structure of the protein (Supplementary Material 2, 3). The PROMOTIF module of this program showed that the functional protein contains 316 residues among which 114 (36.1% of the residues) form strands, 15 (4.7%) alpha-helices. The remaining 187 (59.2%) form either β -hairpins, α- or β -sheets and bulges or one γ-turn. A predicted 3D-model of the protein was also established from this analysis (Supplementary Material 4). The same program generates also a PROCHECK analysis of the protein. The Ramachandran plot (Ramachandran et al., 1963) revealed a significant 84.6% of residues in most favored regions within the phipsi core [A,B,L] (Supplementary Material 5). Additional analysis included in the ProFunc reports such as InterPro scan (Quevillon et al., 2005) of the sequence motifs and the search of the sequence against superfamily HMM library (Finn et al., 2006) confirmed once again the presence of a Cupin_2 motif and the belonging to the RmlC-like cupin superfamily (Supplementary Material 6).

Sequence matching against existing PDB entries revealed certainty matches (*E-value < 1.00e-06*) with quercetin 2,3-dioxygenases from *A. japonicus* and *B. subtilis*. BLAST search against Uniprot database showed also certainty matches (*E-value <1.00e-06*) with dioxygenases from *A. fimugatus, A. flavus*, and proteins from *P. chrysogenum* and *Neosartorya fisheri*, both belonging to the cupin superfamily. In terms of ligand-binding templates, the analysis showed 12 significant hits, two of which were probable (*1.00e-06 < E-value < 0.01*) with “TLAc0047” from *B. subtilis* and “_CUc0024” from *A. japonicas*, and one possible match (*0.01 < E-value < 0.1*) with “_CUc0075” form *A. japonicus*. The reverse template comparison *vs*. the structures in PDB revealed a certainty match (*E-value < 1.00e-06*) with “pdb_1juh”, which is the quercetin 2,3-dioxygenases from *A. japonicus* (Supplementary Material Xls file 1).

**Figure 3:**
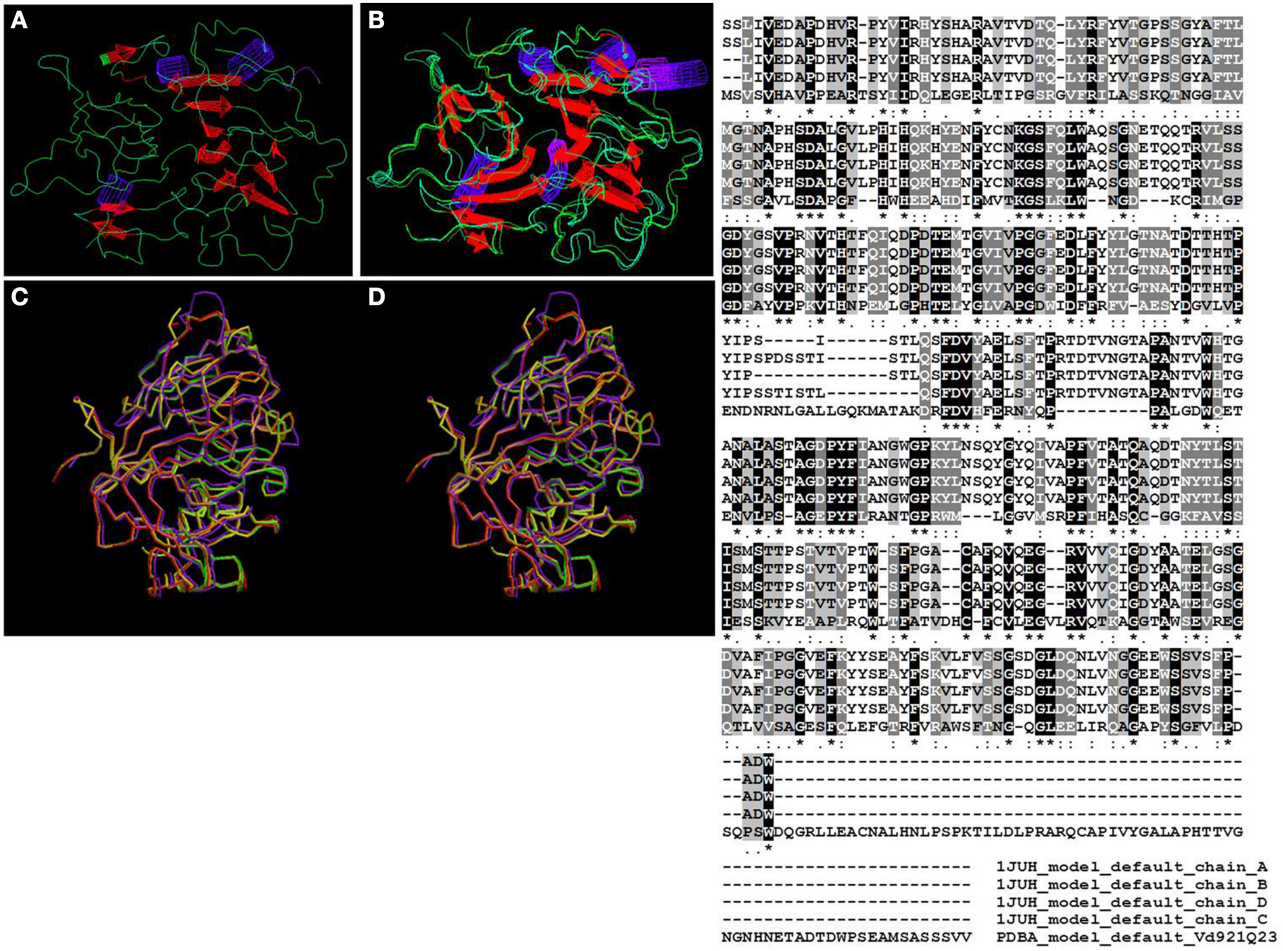
**Theoretical model generated using WhatIf program illustrating the structure of the quercetinase from** ***V. dahliae*** **(A) and its superposition with the template quercetin 2,3-dioxygenase from** ***A. japonicus*** **(B). (C, D)** are outputs of the SuperPose program, where **(D)** represents the *V. dahliae* protein and **(C)** the the template quercetin 2,3-dioxygenase from *A. japonicus*. Minor conformational changes can be noticed between the two structures. The ClustalW2 alignment conducted by the SuperPose program during the superposition of the quercetinase protein from *V. dahliae* (PDBA_Vd921Q23) and the template quercetin 2,3-dioxygenase from *A. japonicus* (1JUH_), which have four chains labeled **(A–D)** highlights the high degree of conservation between the two structures. “^*^” means that the residues in the column are identical in all aligned sequences; “:” means that conserved substitutions have been observed; “.” means that semi-conserved substitutions are observed; “-” means that there is a gap between the aligned sequences.

Further analysis using WhatIf (Vriend, 1990) and SuperPose (Maiti et al., 2004) programs allowed for the superposition of the 3D-predicted structure of the protein with the certainty template match “pdb_1juh.” This along with the generated ClustalW analysis of the aligned sequences from the template revealed a high degree of conservation as well as conserved and semi-conserved substitution in the amino-acid sequences (Figure 3).

### *In vitro* assay of the enzymatic activity

**Figure 4:**
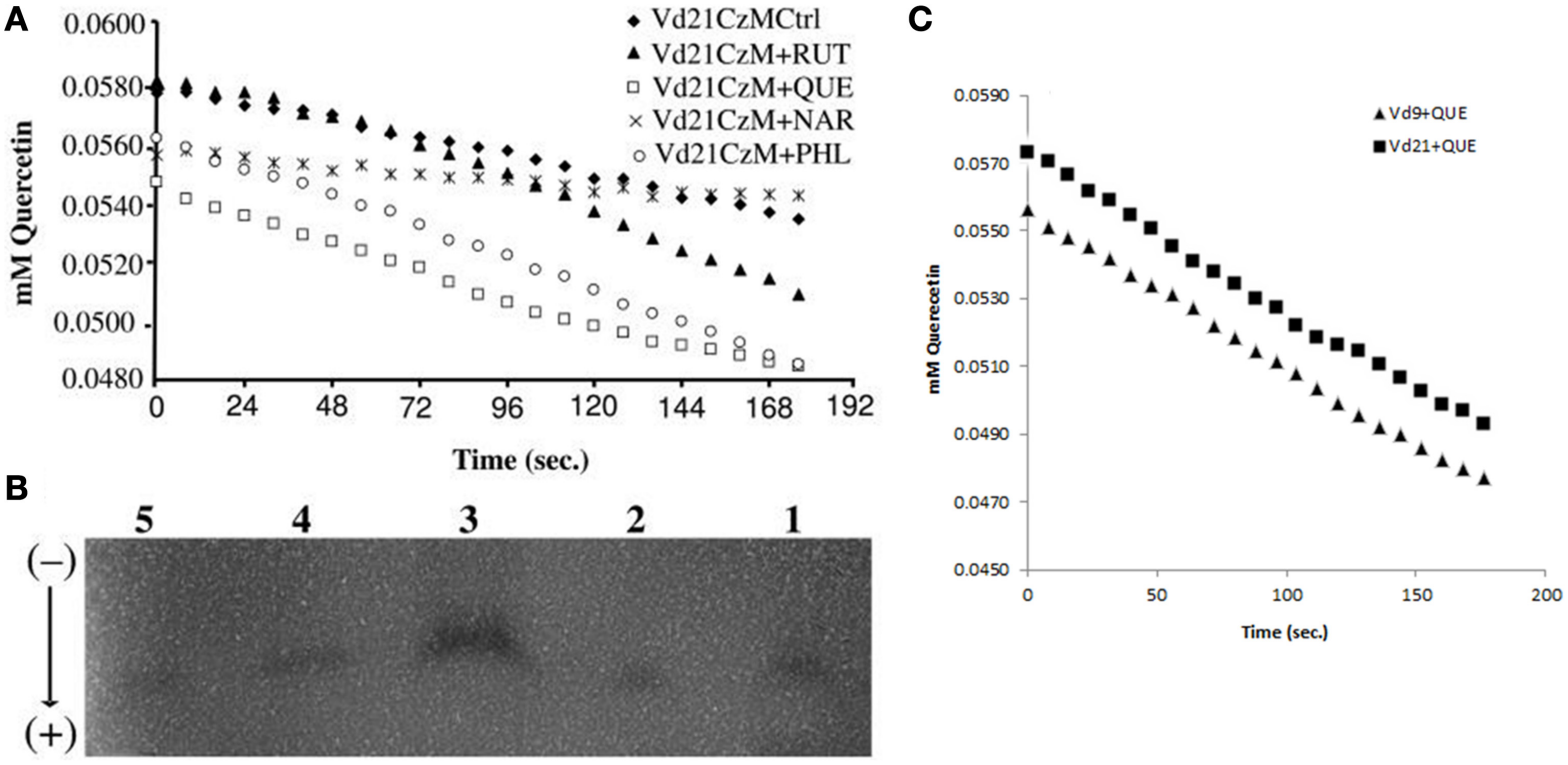
**(A)** Quercetinase activity form *V. dahliae* isolate Vd21 grown on Czapeck-Dox liquid media (CzM) control (Ctrl, 1) or amended with rutin (RUT, 2), quercetin (QUE, 3), naringenin (NAR, 4), or phloroglucinol (PHL, 5), **(B)** isoforms detected on horizontal cellulose acetate electrophoresis system (Helena Laboratories, Beaumont, TX) and **(C)** comparison of the activity of the VdQase in the two tested isolates Vd9 and Vd21.

The examination of quercetinase *in vitro* (Figure 4A) revealed an activity of 160 IU; one IU being 1 μmole of quercetin cleaved per hour under standard conditions, with a *Km* ranging from 0.0025–0.0036 for quercetin (calculated in μmol quercetin min^−1^) and 0.06–0.08 for O_2_ (estimated). The specific activity average was 2516 μmol quercetin min^−1^ mg^−1^ protein g^−1^ mycelium FW. The use of dioxygenase inhibitors such as kojic acid or prohexadione-Ca abolished the quercetinase activity (El Hadrami et al., 2011). Similarly, using chelated copper (Cu-EDTA) did not yield any enzymatic activity (data not shown).

The separation of *V. dahliae* quercetinase isozymes on horizontal cellulose acetate gel electrophoresis showed at least three isoforms of the enzyme when the proteins were extracted from mycelia grown on liquid media amended with rutin, quercetin or naringenin. When the mycelia were collected from the control or from media amended with phloroglucinol, only one isoform was detected (Figure 4B). The overall activity of the quercetinase was similar among the two tested isolates Vd9 and Vd21 (Figure 4C).

### Analyses of the mutagenesis of VdQase gene

**Figure 5:**
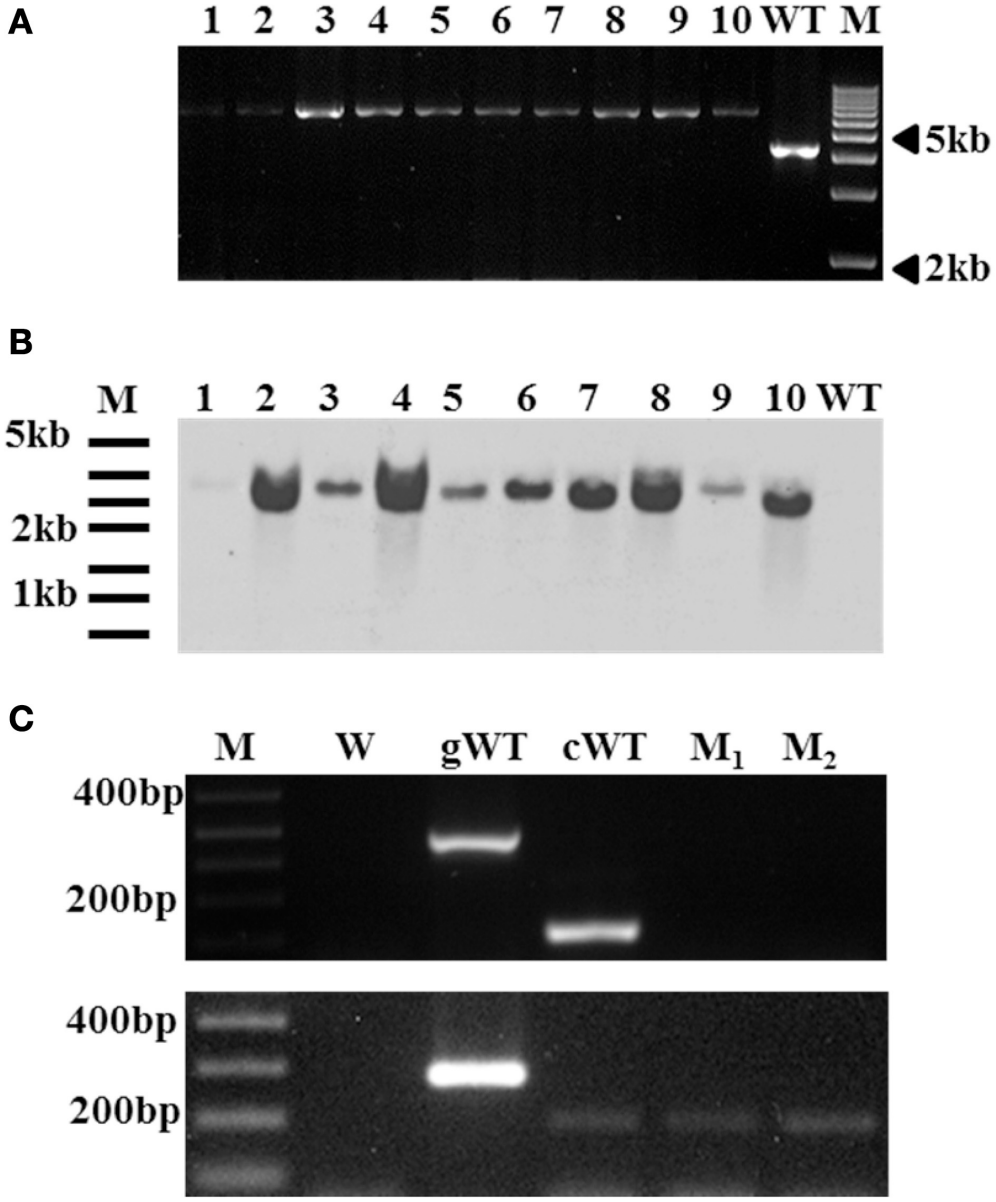
**Confirmation of** ***VdQase*** **mutant transformants by PCR, southern hybridization and RT-PCR analyses. (A)** PCR confirmation of the double homologous recombination in the VdQase mutant by the replacement of wild type copy with mutant allele using a primer designed based on few hundred bp upstream sequence from the start codon and a VdQase gene specific reverse primer. M-Marker, WT-Wild type, Mutants (1–10). **(B)** Southern hybridization analyses of the wild type and the mutants with *EcoR*I digested genomic DNA and *hygromycin B* as a probe. M-Marker, WT-Wild type, Mutants (1–10). **(C)** RT-PCR analyses. M-Marker, W-Water control, gWT-Genomic DNA wild type, cWT-cDNA wild type, M1-Vd9+25-5 (Mutant 1) and M2-Vd9+25-7 (Mutant 2). The lower panel shows the expression of Actin in the wild type (both genomic and cDNA) and the VdQase mutant strains (M1 and M2).

*VdQase* gene replacement mutant was produced by ATMT of the mutagenized gene into isolate Vd9. Sequence analyses of the mutagenized plasmids (pDHtVdQaseTn25) confirmed the insertion of EZ::TN after the 355th base of the second intron of the *VdQase* coding sequence. PCR analysis confirmed the replacement of the wild type copy of *VdQase* gene in the mutant transformants (Figure 5A). Southern blot analyses confirmed the insertion of a single copy of the hygromycin marker gene (Figure 5B). Two of the analyzed transformants (Figure 5C), Vd9+25-5 (M1), Vd9+25-7 (M2), were selected for further analyses based on their morphology, growth rate and spore production that were similar to the wild type.

### VdQase is involved in pathogenicity

**Figure 6:**
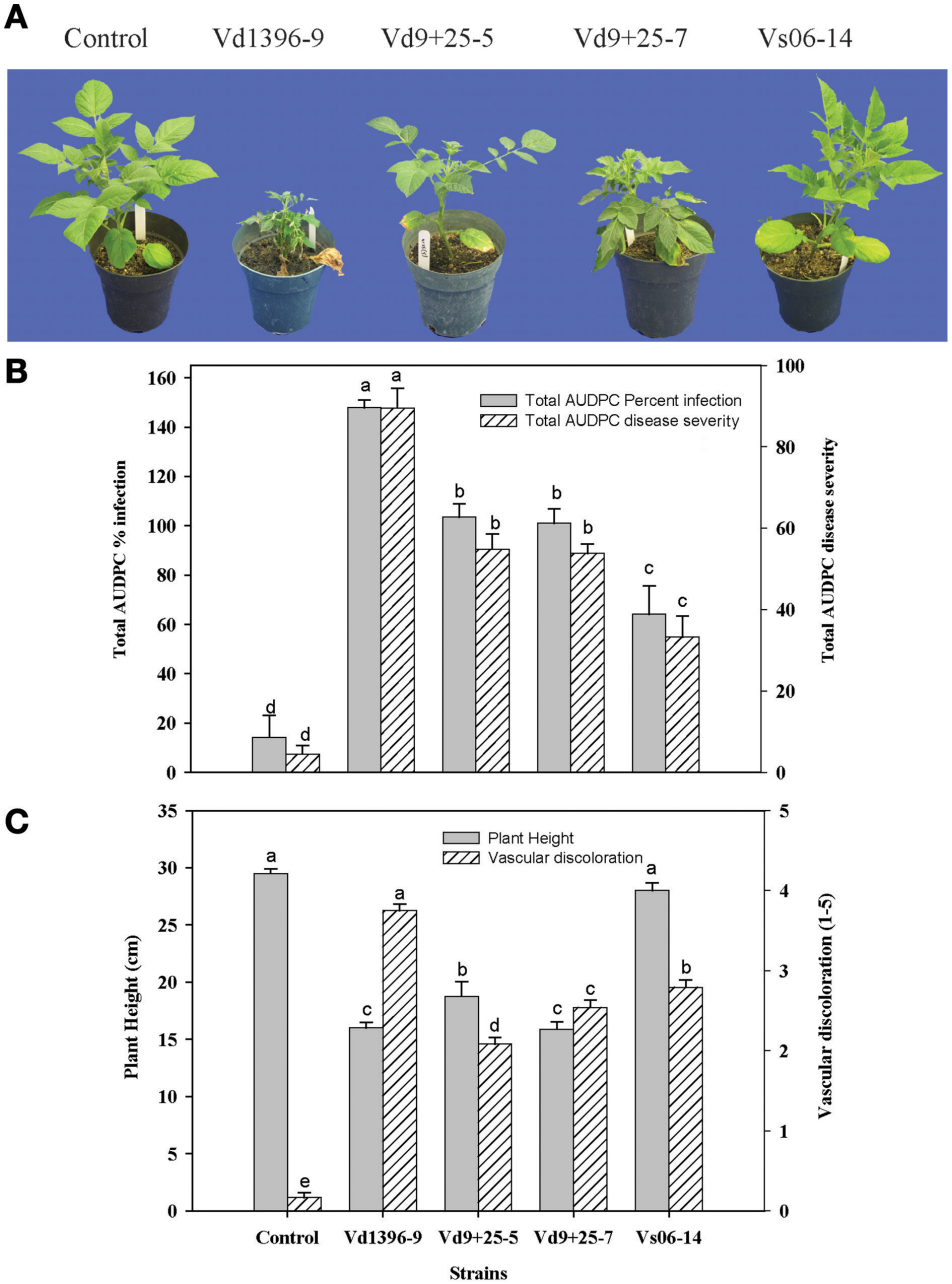
**Pathogenicity assessment of** ***V. dahliae*** **quercetinase (VdQase) mutants on potato. (A)** Symptoms on susceptible potato cv. Kennebec, **(B)** Per cent AUDPC plant infection and AUDPC disease severity, and **(C)** Plant height and stem vascular discoloration of inoculated plants. Total AUDPC (plant infection and disease severity) values were calculated based on data collected at 2, 3, and 4 week post-inoculation. Control (non-inoculated), Vd9 (highly aggressive wild type isolate), Vs06-14 (weakly aggressive wild type isolate), Vd9+25-5 (VdQase mutant 1), and Vd9+25-7 (VdQase mutant 2). Columns with the same letters do not differ significantly according to Newmann-Keuls test at *P* < 0.05. Bars with the same letter are not significantly different according to Duncans multiple range test (α = 0.05).

The findings of the present study along with our previous findings clearly indicated the ability of *V. dahliae* to metabolize the flavonol compound rutin due to its glucosidases and rhamnosidases which cleave the sugar moieties of this flavonol compound, thereby freeing the quercetin aglycone. The extracellularly-secreted quercetinases would break down quercetin and release a bi-product protocatechuic acid (PA). It was hypothesized that *V. dahliae* quercetinases may be involved in the pathogenicity of this soil-borne pathogen through utilization of the flavonol compound rutin. To test this hypothesis, we constructed a knock-out insertional mutant. The results revealed that *VdQase* mutants display reduced pathogenicity in potatoes as compared to the wild type isolate Vd9 (Figure 6). While the wild-type isolate produced severe chlorosis and wilting symptoms on the infected plants, the mutants produced very few wilt symptoms during the entire period of the assay. Interestingly, the *VdQase* mutants were more aggressive than weakly aggressive isolate Vs06-14.

Both AUDPC % infection and severity were lower with the VdQase mutants than with the wild type aggressive isolate (Vd9), whereas the mutants had higher aggressiveness compared to the weakly aggressive isolate (Vs06-14). Stunting and vascular discoloration symptoms caused by the mutants were less pronounced than in response to either wild type highly aggressive isolate Vd9 or weakly aggressive isolate Vs06-14.

### Role of VdQase in rutin and quercetin utilization

**Figure 7:**
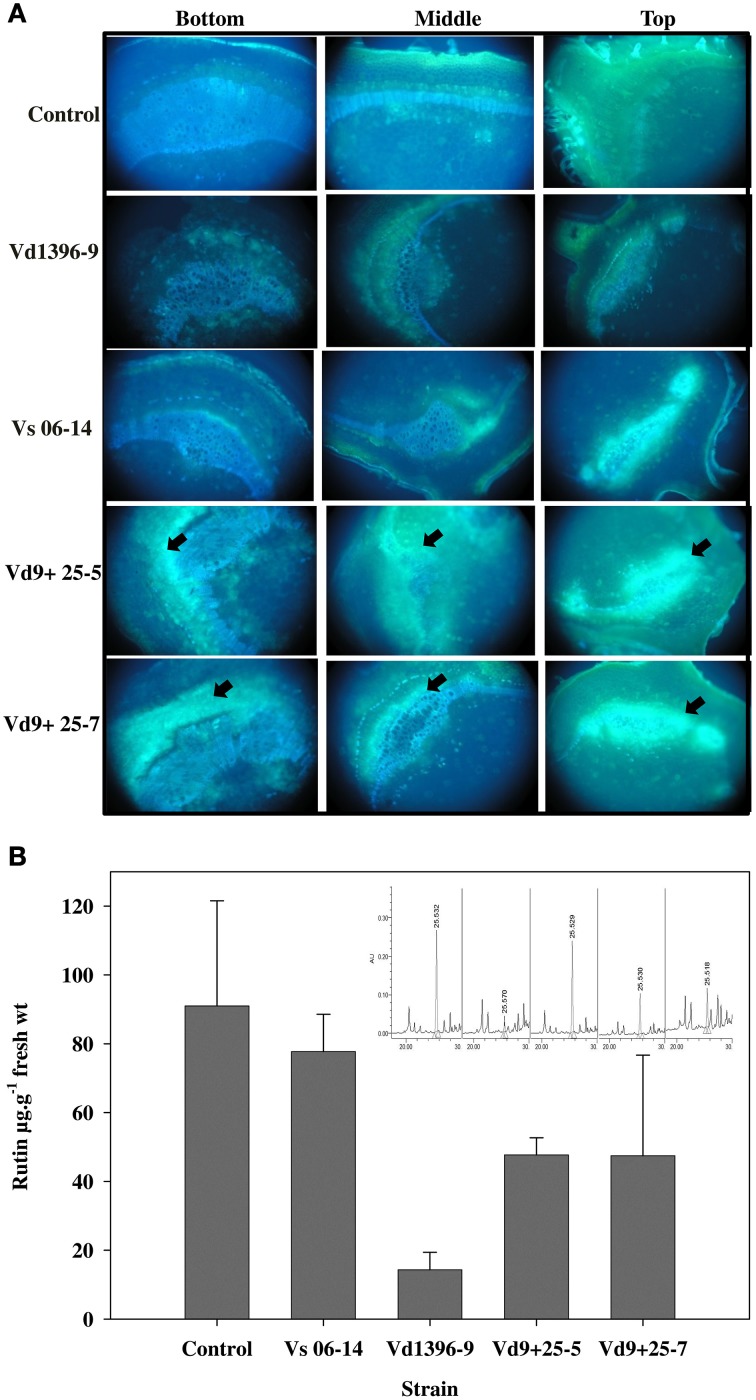
**Utilization of flavonol compounds by** ***V. dahliae*** **quercetinase (VdQase) mutants** ***in planta***. **(A)** Fluorescence microscopy showing the accumulation of the flavonol compounds in the potato stem infected with *V. dahliae* wild type and the mutants at 28 dpi as indicated by the yellow fluorescence detected by immersing the cross sectioned potato stem in 1% Neu's reagent. **(B)** *In planta* utilization of rutin by VdQase mutant as quantified by HPLC. HPLC chromatograms were shown at the top right corner for rutin. Control (non-inoculated), Vd9 (highly aggressive wild type), Vs06-14 (a weakly aggressive wild type), Vd9+25-5 (VdQase mutant 1), and Vd9+25-7 (VdQase mutant 2). Arrows indicate the accumulation of flavonoids including rutin and quercetin in potato tissues.

To confirm our hypotheses regarding the utilization of the flavonol compound rutin and its break down product quercetin by *V. dahliae*, we first aimed to assess the flavonol compounds qualitatively using fluoresecence microscopy with the potato stem sections inoculated with wild type or *VdQase* mutants. The use of fluorescence microscopy to assay rutin utilization *in planta* revealed brighter yellow florescence in the tissues infected with the VdQase mutants as compared to the wild type and the non-inoculated control (Figure 7A). The fluorescence intensity in response to the mutant was similar to the weakly aggressive isolate Vs06-14 (Figure 7A). These results primarily suggest the involvement of VdQase in metabolism of the flavonol compound rutin. To unravel the facts of rutin utilization by *V. dahliae* by the extracellularly secreted quercetinases, the wild type and the mutant strains were grown in CDX medium supplemented with rutin and quercetin. A lower percentage of non-utilized rutin was recorded in presence of the highly aggressive wild type as compared to the VdQase mutants and the weakly aggressive wild type *in planta* (Figure 7B).

### Role of the VdQase in the manipulation of the host SA- and JA-signaling pathways

**Figure 8:**
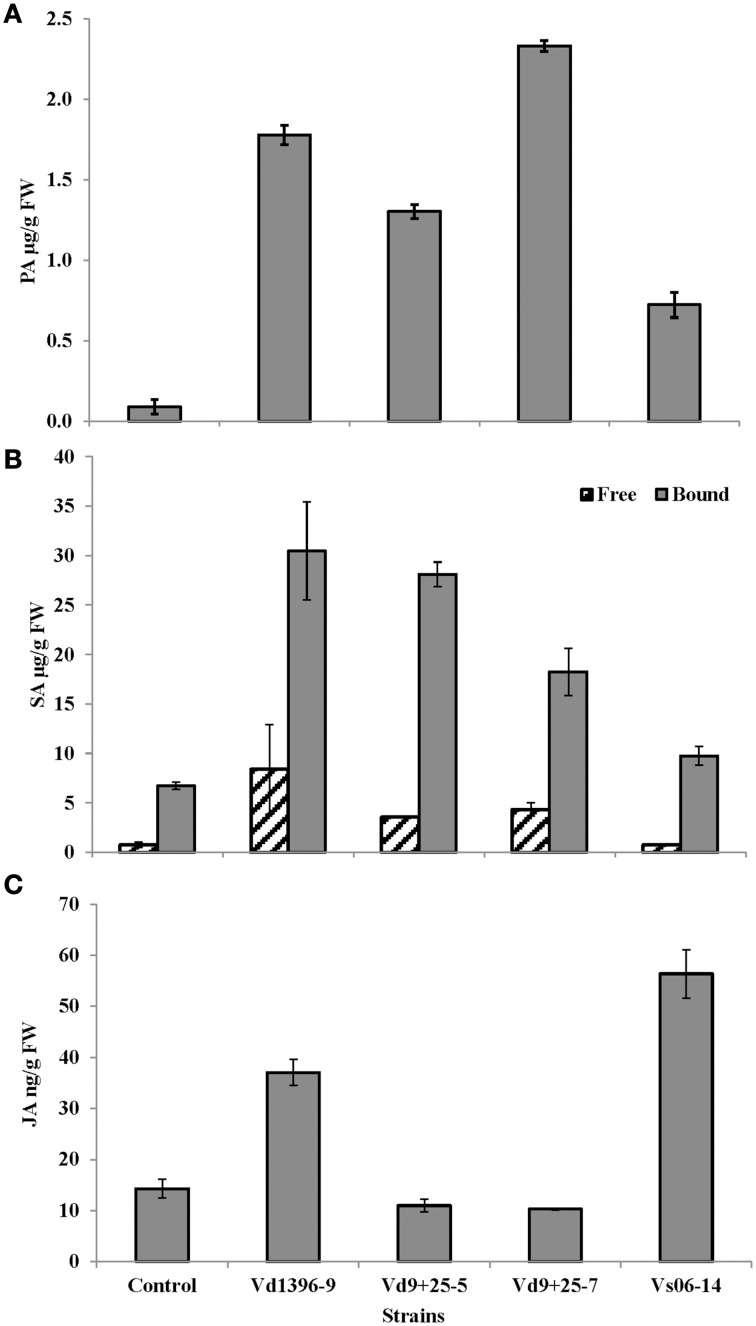
**HPLC quantification of (A): protocatechuic acid (PA), (B): free and bound salicylic acid (SA), and (C): Jasmonic acid (JA), in potato leaves 28 days after inoculation with different** ***V. dahliae*** **isolates: highly aggressive wild type isolate Vd9, weakly aggressive wild type isolate Vs06-14, quercetinase (VdQase) mutant1 Vd9+25-5, or mutant2 Vd9+25-7. Control is the non-inoculated check**.

To address the mechanisms how *V. dahliae* overcome the host defenses to regulate its pathogenicity, we quantified the PA along with the end products SA and JA in the tissues infected with the wild type and the mutant strains. Protocatechuic acid, a by-product of rutin catabolism, had higher levels in tissues infected with the highly-aggressive wild type isolate Vd9 and the *VdQase* mutants as compared to the weakly-aggressive wild type isolate Vs06-14 or the untreated control (Figure 8A). Levels of free and bound SA were higher in tissues infected with the wild type highly-aggressive isolate Vd9 as compared to the *VdQase* mutants and the weakly-aggressive wild type Vs06-14 (Figure 8B). Levels of free and bound SA were also two to three times higher in tissues inoculated with the highly aggressive wild type isolate Vd-9 as compared to the VdQase mutants (Figure 8B). However, SA levels in response to the VdQase mutants were higher than those in response to the weakly aggressive wild type Vs06-14. Quantification of JA revealed higher levels in potato leaves inoculated with the weakly aggressive isolate Vs06-14 as compared to highly aggressive wild type Vd9 (Figure 8C). JA levels in response to the VdQase mutants were lower than both the weakly- and highly-aggressive wild type isolates.

## Discussion

In the current study, we have characterized a cupin domain-containing protein with a quercetinase activity (VdQase) that seems to regulate *V. dahliae*'s pathogenicity and contribute to its ability to counteract host defenses. Three primer sets designed in the conserved regions of known quercetinases from *P. olsonii, A. japonicus* and *Streptomyces* sp. strain FLA were used to amplify putative quercetinase-coding sequences in *V. dahliae*. Sequenced products were BLAST-searched against multiple databases and an ORF was located in the annotated *V. dahliae* genome available on MIT website (www.broadmit.edu/). Full-length transcripts were then isolated from mycelia incubated in presence of rutin or quercetin as well as other related flavonones (naringenin). A series of *in silico* analyses was conducted using amplified and sequenced *V. dahliae* products from the conserved region or the full length to determine various properties of each sequence. An *in vitro* analysis of the enzymatic activity followed to establish its role in cleaving quercetin. Furthermore, a mutagenesis approach using gene replacement in a highly-aggressive isolate Vd9 was used to establish the role of this gene in pathogenicity.

The *V. dahliae* sequences amplified using primer sets designed in the conserved regions of known quercetinases (QueVd2F/R, QueVd3F/R) showed significant hits with copper binding domains from the quercetin 2,3-dioxygenase of *A. japonicus* (*E-value* ranging from *1.00e^−06^* to *0.01*). Against the annotated *V. dahliae* genome the sequences showed 85–100% identity with cupin 1, Zn finger C2H2 transcripts fully sequenced and annotated with no assigned function located on chromosome 3 of the two *V. dahliae* and *V. albo-atrum* reference isolates in the database. The ORF was then named VdQase.

Quercetin 2,3-dioxygenase (Q2,3D) was shown to be the only dioxygenase that functions as a homodimer and unequivocally depend on two moles of Cu^2+^ (Grotewold, 2006; Merkens et al., 2007; El Hadrami et al., 2009, 2011). Our investigation of the enzymatic activity of the purified protein showed a dependency on copper as a metal co-factor. Knowing that the mixture flavonol-Cu^2+^ can cause damage to fungal DNA in absence of reducing agents (Ahmed et al., 1994; Phillips, 1993), it is apparent that *V. dahliae*'s quercetinase plays an important role in countering the fungitoxic secondary metabolites produced by the host plant (i.e., rutin, quercetin). The action of this enzyme is not restricted to quercetin since it was found to act on several related flavonols. However, the *Km* and *Vmax* of this enzyme seem to depend on the OH topology at the A and B rings (Oka et al., 1972). In the present study, we showed the ability of the same enzyme to cleave other related flavonones (i.e., naringenin).

Sequences amplified using Q2FLAVd2F/R primers set showed, on the other hand, similarities with membrane permeases, sugar transporters/permeases, structural and transmembrane proteins, strongly suggesting a role as structural transmembrane proteins (cupin) with multiple functions, among which dioxygenation of flavnols/ones, i.e., querecetin and naringenin. The latter reaction seems to be favored by an acidification of the plasma membrane and the activity of an ATPase, as well as sugars binding/carrier motifs (i.e., ABC transporter, maltose permeases). This resembles what was described in animal and human cells in presence of a leukemia virus where sugar, quercetin and other flavonones were passively transported through the same transporters (Cunningham et al., 2006). It also agrees with other findings supporting a closer relationship between pathogenicity-related genes and those involved in the primary metabolism in Dothideomycetes i.e., *Stagnospora nodurum* and *Leptosphaeria maculans* (Rahman et al., 1989; Schoefer et al., 2003; Simpson et al., 1960).

Full-length transcripts of VdQase(s) were isolated from mycelia incubated in presence of rutin or quercetin as well as other related flavonones (naringenin). Two homolog genes 1.5 and 2.2 Kb in size were amplified using primer sets Vd23F/R1 and FLAVd2F/R1, respectively, suggesting the presence of multiple quercetinases in *V. dahliae*. The analysis of these transcripts revealed a strong homology and 24–26% identity with RmlC-like Cupin from *A. japonicus* or *B. subtilis*; both known as Quercetin 2,3-dioxygenase-like proteins. A series of *in silico* annotations and analysis including the generation of a PDB signature of the predicted proteins revealed with certainty matches (*E-value < 1.00e-06*) with quercetin 2,3-dioxygenases from *A. japonicus* and *B. subtilis*. In addition, the *in vitro* analysis of the enzymatic activity confirmed its role in cleaving quercetin and established the characteristics of this enzyme. This confirms earlier data showing the detection of the by-product of detoxification of quercetin by UPLC-MS/MS (El Hadrami et al., 2011).

To tentatively establish a role of VdQase in *V. dahliae* pathogenicity, a gene replacement mutagenesis approach was used in a highly-aggressive isolate Vd9. Mutants with single copies of the hygromycin marker gene were successfully generated. Two of them that did not show any impairment in morphology, growth rate or spore production as compared to the wild type were further used to inoculate potato plants and assess disease progress as well as accumulation of rutin *in planta*. The *VdQase* mutants exhibited an attenuated pathogenicity as compared to their counterpart wild type and highly aggressive isolate Vd9, suggesting that VdQase is a pathogenicity-related gene that plays a key role in the expression of Verticillium wilt. However, disease severity caused by these mutants was higher than that of the wild type and weakly aggressive isolate Vs06-14. This suggests either (i) an absence of VdQase in Vs06-14 or (ii) the amount of rutin induced *in planta* upon infection with Vs06-14 is not enough to induce the expression of the VdQase. An alternative hypothesis would be related to the fact that multiple putative VdQases were detectable in *V. dahliae*, allowing the generated mutants to have an intermediate phenotype and to be more aggressive than the weakly-aggressive and wild type isolate Vs06-14.

**Figure 9:**
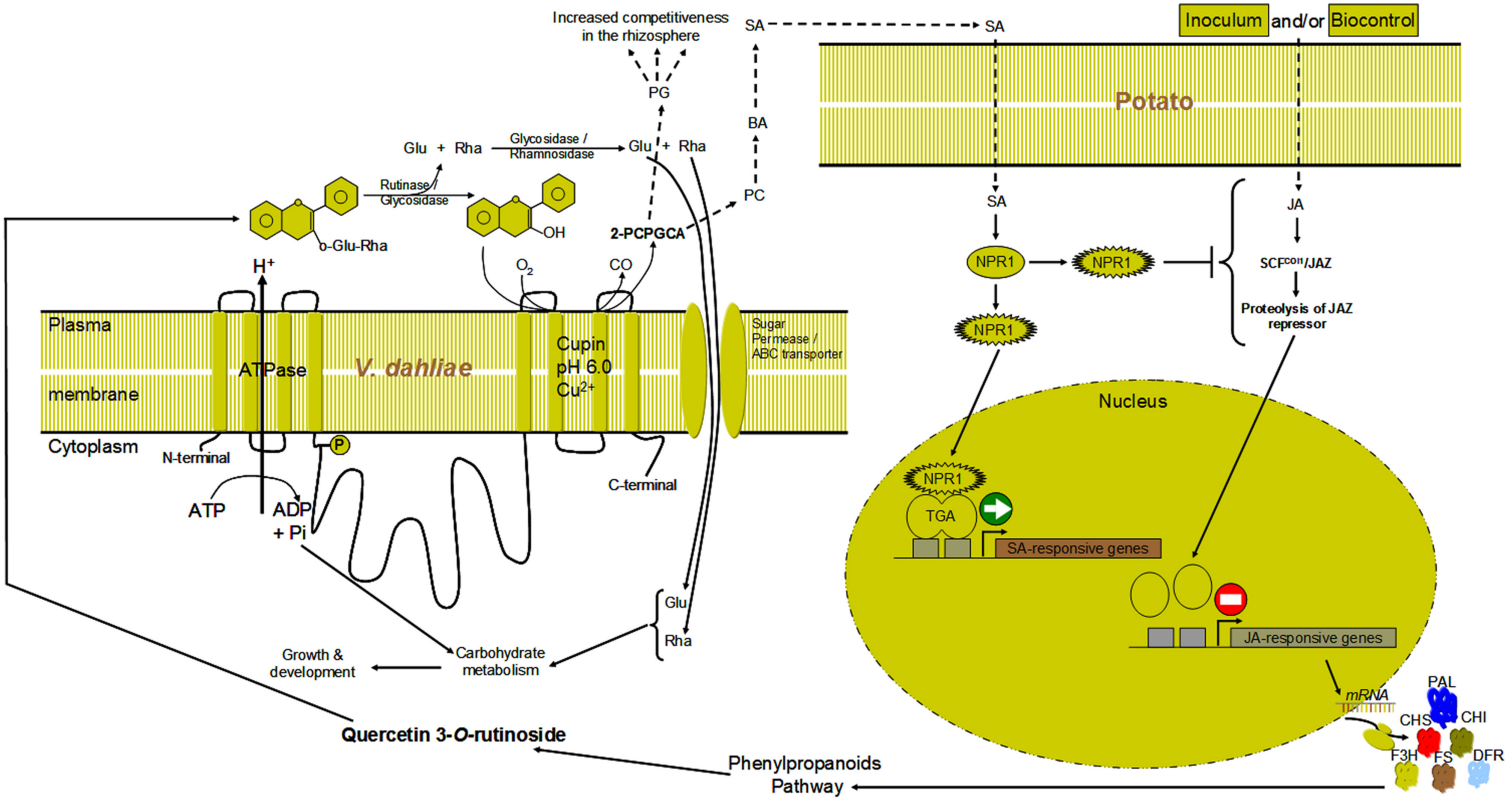
**Hypothetical model illustrating the cellular localization and the link of** ***V. dahliae*** **quercetinase with structural transmembrane proteins, ATPase, other carbohydrate hydrolases and sugar transporters/permeases**. After cleavage of the sugar moieties of rutin a dioxygenation of the aglycone quercetin occurs via the activity of the quercetinase. The by-product of dioxygenation 2-protocatechoylphlorogluciol carboxylic acid (2-PCPGCA) could be cleaved into phloroglucinol (PG) and protocatechuic acid (PC). PC could be converted to benzoic (BA) and salicylic acid (SA). The SA could trigger SA-pathway to interact with the JA via yet unidentified factor(s).

Knowing from an earlier study (El Hadrami et al., 2011) that rutin is the main secondary metabolites induced in potato to a high level (>100 μM) by successful bicontrol agents and only weakly by the non-effective agents, and having characterized in the present study *V. dahliae*' enzyme responsible for its catabolism, it appears that the success of *V. dahliae* in the rhizosphere is partly enhanced by its ability to exhibit a quercetinase activity that allows it to counter host plants' defenses (i.e., synthesis/accumulation of flavonols/ones), turning them into by-products such as 2-PCPGCA (Supplementary Material 1). The latter may play a role as a defensive or an offensive effector in *V. dahliae* counter-defenses/pathogenicity either directly or indirectly. The phloroglucinol moiety as an antibiotic, may, when released, create disequilibrium among the microbial communities including pathogens and antagonists, thereby helping *V. dahliae*'s notoriety in the rhizosphere. The protocatechuic part can be converted to benzoate and salicylates, which can induce SA-related defenses *in planta* (Figure 9). This may interfere indirectly with JA-related potato-defenses against *V. dahliae*, in line with the negative feedback between SA and JA pathways (Derksen et al., 2013). The quantification of PA, SA, JA *in planta* revealed higher levels of PA and SA in response to highly-aggressive and wild type isolate Vd9 as well as the mutants as compared to the control of the weakly-aggressive and wild type isolate Vs06-14. Oppositely, JA content were higher in response to weakly-aggressive and wild type isolate Vs06-14 as compared to highly-aggressive and wild type isolate Vd9 and the mutants. This supports our data from an earlier study showing that Canada milkvetch produces jasmonate-related compounds and mediated JA-related defenses in infected potato plants (El Hadrami et al., 2011).

*VdQase* mutants exhibited lower pathogenicity than the highly aggressive wild type isolate Vd9 and a lower utilization of rutin and quercetin both *in vitro* and *in planta*, suggesting an involvement of this enzyme in key steps of the plant x pathogen interactions. That is the catabolic detoxification of quercetin through dioxygenation. Detection of the protocatechuic acid and phloroglucinol, end products of rutin/quercetin catabolism, confirmed the activity of this enzyme. Assessment of SA and JA revealed lower inductions in response to the *VdQase* mutants and the weakly aggressive wild type isolate Vs06-14 as compared to the wild type highly aggressive isolate Vd9. This confirms our earlier report on the action of rutin in a dose response manner. Higher JA triggers higher synthesis and accumulation of rutin (>100 μM), which is detoxified by the VdQase of highly aggressive wild type isolate. The consequence of such a detoxification is the elicitation of SA pathway (higher free SA). *VdQase* mutants induce lower levels of JA, leading to a low synthesis and accumulation of rutin, which is either not enough to induce the VdQase activity or generate only low levels of SA to interfere with the JA pathway. Elicitation of one pathway (i.e., SA-pathway) to hijack another pathway that is more adequate for defense (i.e., JA-pathway) had similarly been described in tobacco in response to *Pseudomonas syringae* (Rayapuram and Baldwin, 2007), *A. thaliana* in response to *F. oxysporum* (Thatcher et al., 2009) and tomato in response to Botrytis (El Oirdi et al., 2011).

In the present study we were able to characterize *in silico* and *in vitro* the *VdQase* enzyme responsible for the catabolism of the main secondary metabolite induced in potato plant (rutin) in response to infection by *V. dahliae* or elicitation by biocontrol agents. We also generated mutants to functionally confirm the role of this enzyme. Based on an indication that rutin can be transported systemically *in planta* (Buer et al., 2007), a zigzag race (Figure 9) between the pathogen and the host, would lead either to an enhanced host defense if the rutin accumulates at levels high enough to restrict the pathogen's growth and/or development, or to a counter-defense by the pathogen in case this flavonol glycoside accumulates below a certain threshold level. Biocontrol agents to reduce Verticillium wilt in potato could be efficient if they are able to induce this metabolite at higher level but, in the light of the present findings, their application could be managed to alter the zigzag scheme during the interaction.

## Author contributions

All co-authors contributed to the conception of the ideas, design of the experiments, analysis, and data interpretation of different parts of the study. FD supervised the work. AE, MI, and LA run the pathogenicity tests. AE completed the gene expression, *in-silico* annotation, structure and identification. MI generated the VQase mutants and run the functional analysis tests. AE and LA did the chromatography analyses for the identification and quantification of metabolites. AE, MI, and FD wrote the manuscript, and all co-authors reviewed it.

### Conflict of interest statement

The authors declare that the research was conducted in the absence of any commercial or financial relationships that could be construed as a potential conflict of interest.

## Abbreviations

A_367nm_: absorbance at 367 nm
ATMT: *Agrobacterium*-mediated transformation
BA: benzoic acid
DMSO: dimethyl sulfoxide
DPH: 1,6-diphenyl-1,3,5-hexatriene
DTT: Dithiothreitol
FW: fresh weight
IPM: Integrated Pest Management
IU: International unit of an enzyme
JA-pathway: Jasmonic Acid-pathway
LC–MS-MS: Liquid chromatography coupled to mass spectrophotometer
MAFFT: multiple sequence alignment based on fast Fourier transform
MES: 2-(*N*-morpholino)ethanesulfonic acid
MTT: methyl thiazolyl blue
ORF: open reading frame
PMS: phenazine methosulfate
PDA: Potato Dextrose Agar
2-PCPGCA: 2-protocatechuoylphloroglucinolcarboxylic acid
PRRN: progressive pairwise alignment and iterative refinement
SA: salicylic acid
SDW: sterile distilled water
SD-DEPC-W: sterile distilled-DEPC-treated water
UPLC: Ultra Performance Liquid Chromatography.
