# Supplementary figures and images for: A cupin domain-containing protein with a quercetinase activity (VdQase) regulates Verticillium dahliae's pathogenicity and contributes to counteracting host defenses
Source: Front Plant Sci. 2015 Jun 10;6:440. doi: 10.3389/fpls.2015.00440 (PMC4462102; doi:10.3389/fpls.2015.00440)

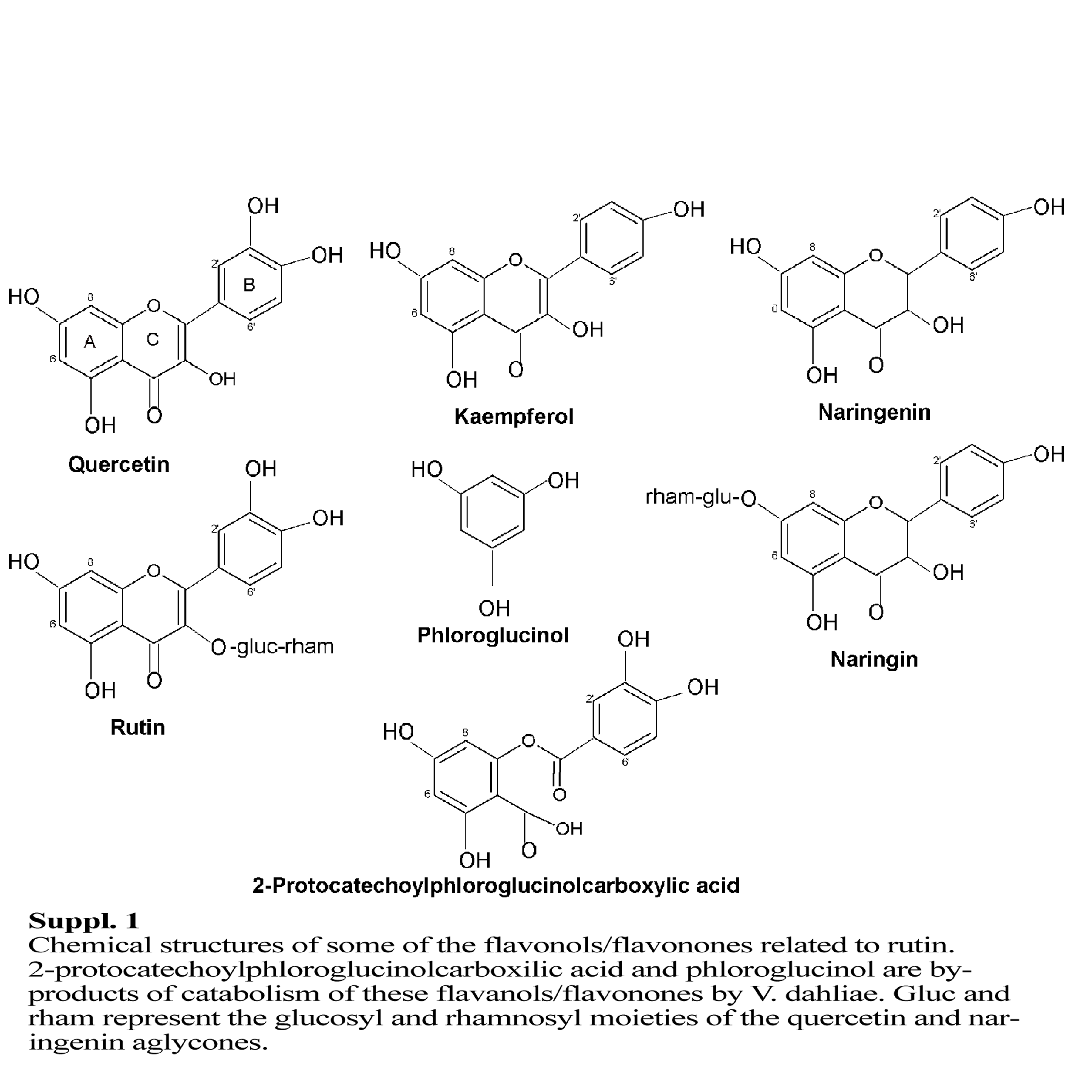

Supplement: Supplementary file 1 [file Image1.TIF]

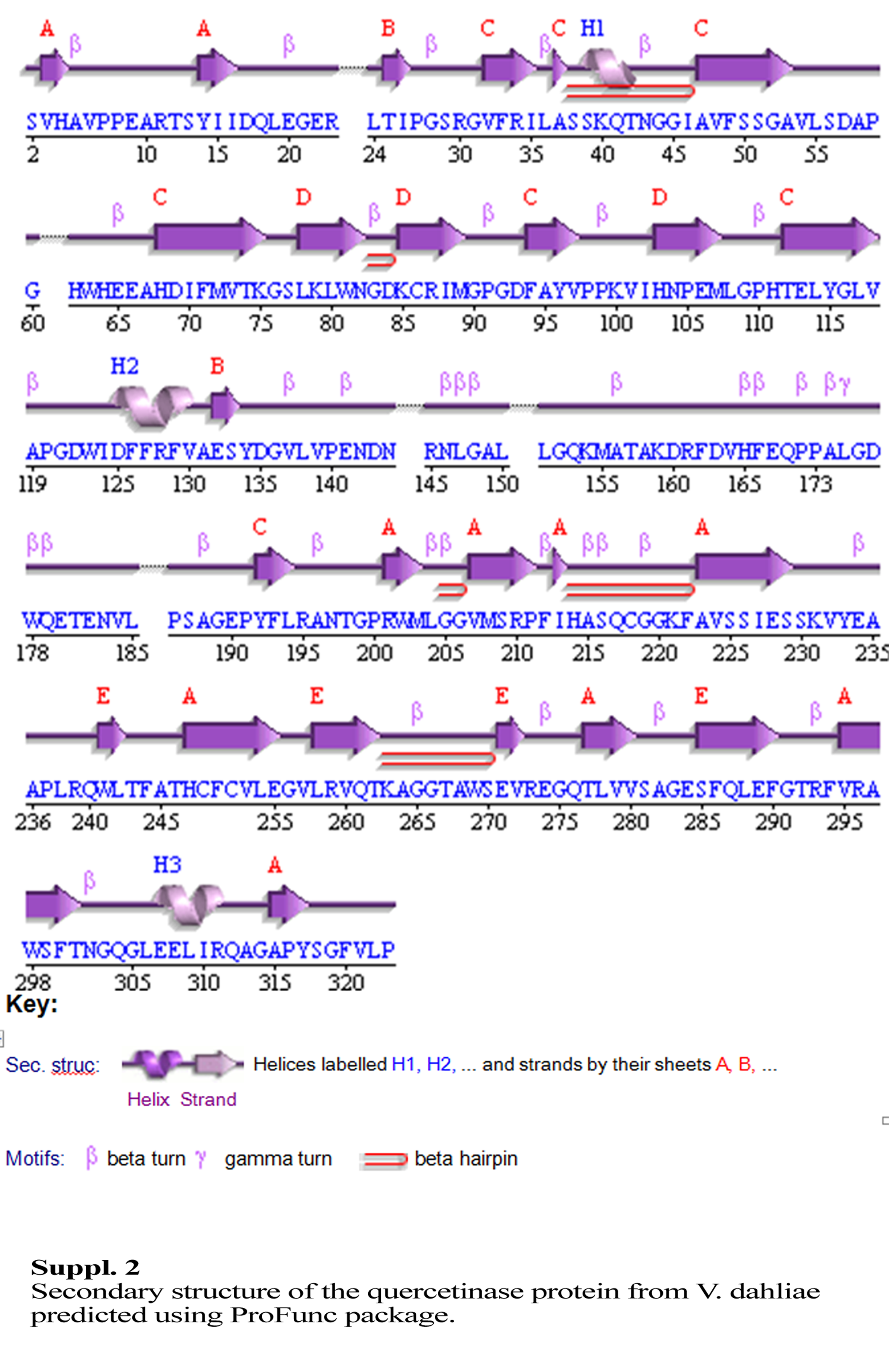

Supplement: Supplementary file 2 [file Image2.TIF]

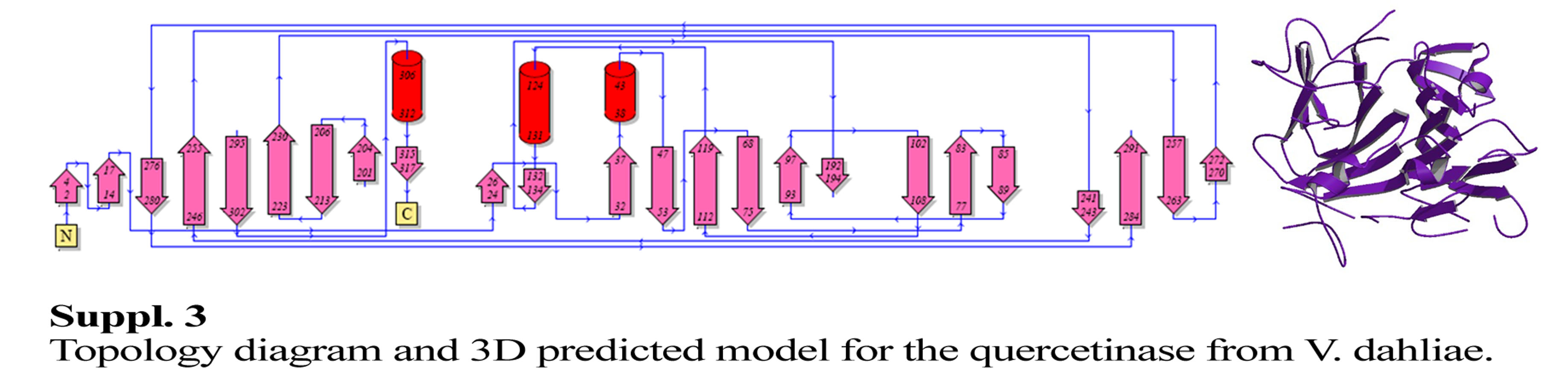

Supplement: Supplementary file 3 [file Image3.TIF]

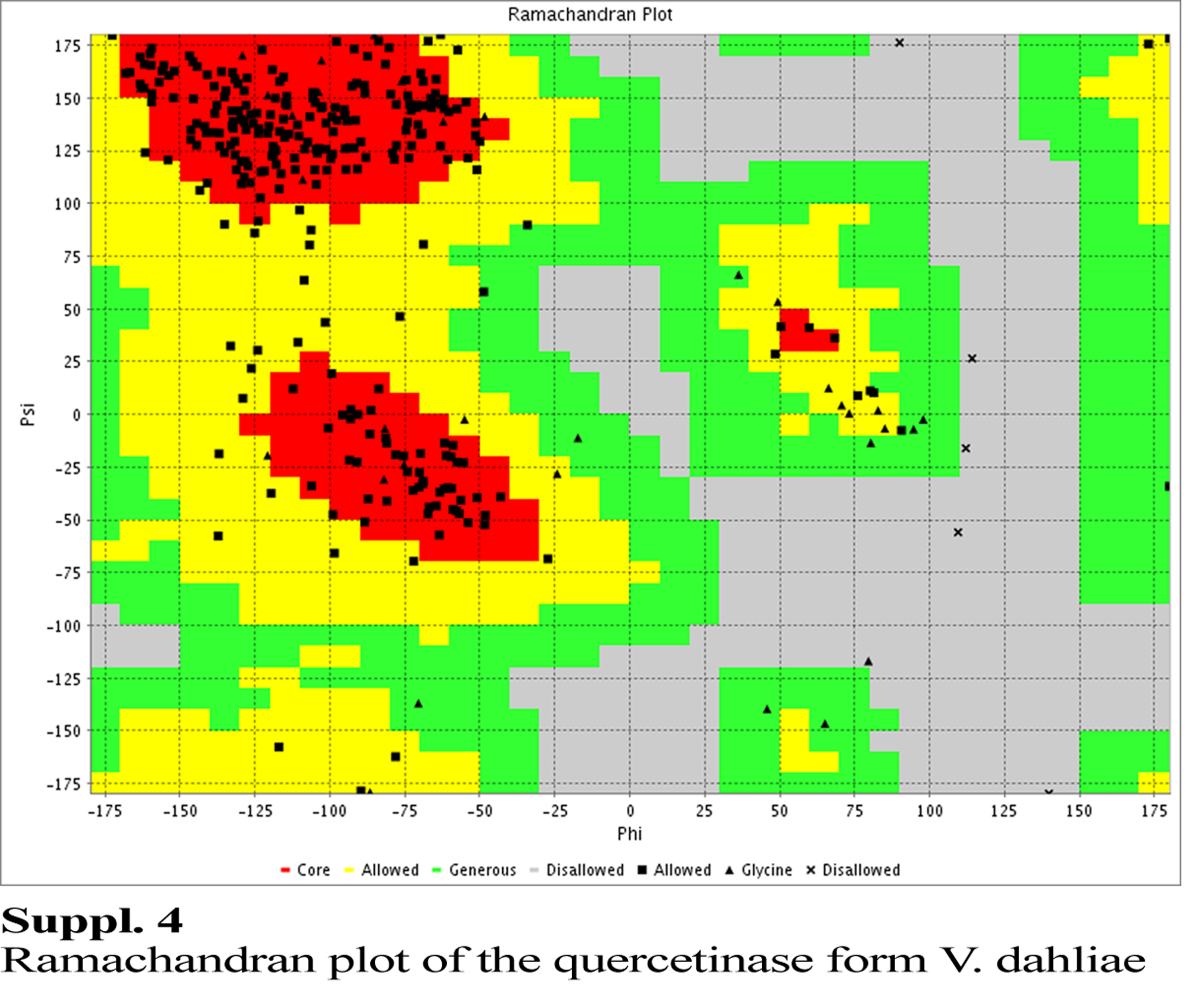

Supplement: Supplementary file 4 [file Image4.TIF]
